# Renoprotective Effects of Maslinic Acid on Experimental Renal Fibrosis in Unilateral Ureteral Obstruction Model via Targeting MyD88

**DOI:** 10.3389/fphar.2021.708575

**Published:** 2021-09-13

**Authors:** Wenjuan Sun, Chang Hyun Byon, Dong Hyun Kim, Hoon In Choi, Jung Sun Park, Soo Yeon Joo, In Jin Kim, Inae Jung, Eun Hui Bae, Seong Kwon Ma, Soo Wan Kim

**Affiliations:** Department of Internal Medicine, Chonnam National University Medical School, Gwangju, South Korea

**Keywords:** maslinic acid, renal interstitial fibrosis, MyD88, Smad4 nuclear expression, unilateral ureteral obstruction, NF-κB

## Abstract

Maslinic acid (MA), also named crategolic acid, is a pentacyclic triterpene extracted from fruits and vegetables. Although various beneficial pharmacological effects of MA have been revealed, its effect on renal fibrosis remains unclear. This study was designed to clarify whether MA could attenuate renal fibrosis and determine the putative underlying molecular mechanisms. We demonstrated that MA-treated mice with unilateral ureteral obstruction (UUO) developed a histological injury of low severity and exhibited downregulated expression of fibrotic markers, including α-smooth muscle actin (α-SMA), vimentin, and fibronectin by 38, 44 and 40%, and upregulated expression of E-cadherin by 70% as compared with untreated UUO mice. Moreover, MA treatment restored the expression levels of α-SMA, connective tissue growth factor, and vimentin to 10, 7.8 and 38% of those induced by transforming growth factor (TGF)-β in NRK49F cells. MA decreased expression of Smad2/3 phosphorylation and Smad4 in UUO kidneys and TGF-β treated NRK49F cells (*p* < 0.05, respectively). Notably, MA specifically interferes with MyD88, an adaptor protein, thereby mitigating Smad4 nuclear expression (*p* < 0.01 compared to TGF-β treated group) and ameliorating renal fibrotic changes (*p* < 0.01 for each fibrotic markers compared to TGF-β induced cells). In addition, in the UUO model and lipopolysaccharide-induced NRK49F cells, MA treatment decreased the expression of IL-1β, TGF-α and MCP-1, ICAM-1, associated with the suppression of NF-κB signaling. These findings suggest that MA is a potential agent that can reduce renal interstitial fibrosis, to some extent, via targeting TGF-β/Smad and MyD88 signaling.

## Introduction

Chronic kidney disease (CKD) is a significant health problem. In recent years, the increasing prevalence of CKD is evident among about 5% of younger adults (aged 20–39 years) as well as nearly half of older adults (aged 70 years and above) (Chou and Chen, 2021). A key step involved in CKD is renal fibrosis, which includes glomerular fibrosis and tubulointerstitial fibrosis. Renal interstitial fibrosis is characterized by the excessive production and deposition of extracellular matrix (ECM) proteins and is strongly correlated with tubulointerstitial inflammation (Liu, 2011; Urban et al., 2015; Humphreys, 2018). This process has been confirmed to involve the following key events: loss of epithelial adhesions, reorganization of the cytoskeleton, *de novo* synthesis of α-smooth muscle actin (α-SMA), destruction of the tubular basement membrane, and enhanced cell migration and interstitial invasion (Geiger and Yamada, 2011; Bülow and Boor, 2019). These events involve changes in the expression of many proteins, such as the early downregulation of E-cadherin expression, which leads to decreased intercellular adhesion, upregulated mesenchymal vimentin expression, and increased protein synthesis such as fibronectin and collagen (I and III) synthesis (Bonnans et al., 2014; Škovierová et al., 2018; Theocharis et al., 2019). In the kidneys, connective tissue growth factor (CTGF) is mainly expressed in parietal epithelial cells, some interstitial cells, and visceral epithelial cells. Transforming growth factor (TGF)-β induces CTGF release, thus enhancing the production of fibronectin, collagen I, and vascular endothelial growth factor (Gupta et al., 2000; Gore-Hyer et al., 2002). Ultimately, all these processes of interstitial fibrosis lead to kidney failure, regardless of the underlying disease.

Unilateral ureteral obstruction (UUO) is a classic and widely used model of experimental renal interstitial fibrosis. This model is established via ligation of one side of the ureter, leading to urinary tract obstruction. The obstruction injury of the kidney is accompanied by tubular atrophy and irreversible interstitial fibrosis, and its hallmark event is the excessive production and deposition of ECM in the interstitium (Popper et al., 2019). ECM proteins are produced and secreted by myofibroblasts, and it has been demonstrated that epithelial-mesenchymal transition (EMT) and interstitial fibroblast activation are two important events for transdifferentiation of myofibroblast cells (Savagner, 2001).

TGF-β is a key mediator driving the fibrotic reaction of most organs (Pohlers et al., 2009). It is synthesized and secreted by inflammatory cells and various effector cells. TGF-β activation leads to the formation and deposition of ECM components; thus, it may become a potential and effective anti-fibrotic target (Chen et al., 2018; Hao et al., 2019). Two signaling pathways are associated with renal fibrosis: Smad as well as non-Smad signaling pathways mediated by TGF-β. In the Smad signaling pathway, binding of TGF-β to its ligand induces the recruitment and phosphorylation of serine-threonine kinases at type I receptors, which in turn phosphorylate Smads (p-Smad2/3). Subsequently, a signaling complex with Smad4 is formed and translocated into the nucleus, thereby upregulating the expression of α-SMA, vimentin, fibronectin, and other ECM proteins or downregulating that of E-cadherin (Wendt et al., 2009; Pardali et al., 2017).

MyD88 is a moderately sized adaptor protein involved in the Toll-like receptor (TLR) and interleukin (IL)-1β receptor signaling pathways involved in mammalian host defense (Brightbill and Modlin, 2000). Most TLR signaling mechanisms mediated by TLR2, TLR5, and TLR9 critically require MyD88 for the initiation of the signaling cascade by assembling a so called “myddosome” high-scaffold signaling complex, which ultimately results in the activation of nuclear factor (NF)-κB (Kawasaki and Kawai, 2014; Chen et al., 2020). Additionally, evidence has indicated that IL-1β can enhance TGF-β signaling and eventually lead to EMT (Artlett, 2012; Zhang et al., 2016). Previous reports suggest that EMT develops as a result of IL-1β binding to its receptors and downstream activation of MyD88 and the c-JUN transcription factor, which could aggravate TGF-β/Smad-mediated fibrosis via increased TGF-β expression (Lee et al., 2006; Jia et al., 2014; Liu X. et al., 2020). Since few studies have focused on the direct association between MyD88 and TGF-β mediated renal fibrosis, further studies are needed to define the functional implications of MyD88 in the TGF-β/Smad signaling pathway. Inflammation has an important role in the development of chronic renal ailments. UUO causes kidney damage primarily through an interstitial inflammatory response, apoptosis, and gradual interstitial fibrosis (Nogueira et al., 2017). Among them, inflammatory reactions are distinguished by an overabundance of cytokines, like pro-inflammasome interleukin (IL)-1β, tumor necrosis factor-α (TNF-α), monocyte chemoattractant protein-1 (MCP-1), and intercellular adhesion molecule-1 (ICAM-1) (Imig and Ryan, 2013; Black et al., 2019).

The NF-κB signaling system is the primary regulator of immunological and inflammatory responses. Also, the activation of NF-κB could result in renal inflammation, tubular EMT, and ECM buildup. (Zavadil and Böttinger, 2005; Tamada et al., 2006). The NF-κB complex is usually inactive and located in the cytoplasm while bound to IκB inhibitor proteins; however, after activation of pro-inflammasome, NF-κB is activated and translocates to the nucleus, where it forms the p65/p50 complex to control target gene transcription (Mussbacher et al., 2019). Lipopolysaccharide (LPS), is well known for its toxicity in the pathogenesis of sepsis in various organs (Kiemer et al., 2002; Hamesch et al., 2015; Liu Z. et al., 2020). Some evidence suggests that renal tubular epithelial cells undergo apoptotic and necrotic processes when LPS-induced inflammation is activated (Bascands et al., 2009). Accordingly, LPS-induced kidney injury may be useful for inflammation associated mechanism study in kidney disease.

MA is a pentacyclic triterpene that occurs naturally in the protective wax-like coating of the leaves and fruit of *Olea europaea L* (Hashmi et al., 2015). Extensive studies have focused on the protective effect of MA against cancer (Reyes-Zurita et al., 2009; Wei et al., 2019; Liu Y. et al., 2020). Furthermore, MA has been confirmed to affect various organs, such as the brain, heart, liver and bones (Qian et al., 2015; Liou et al., 2019; Chen et al., 2021; Shaik et al., 2021). Mechanistic studies have revealed that MA functions in many biological processes, including metabolism, apoptosis, inflammation, and cell proliferation via the NF-κB, PI3K-AKT, and other signaling pathways (Fukumitsu et al., 2016; Liu et al., 2018; Bae et al., 2020; Lee et al., 2020). Especially, Liu et al. found that MA treatment inhibits cardiac hypertrophy and cardiac fibrosis in mice via reduced phosphorylation of ERK and AKT signaling molecules (Liu et al., 2018). Also, researchers demonstrated that MA improves oxidative stress and renal function in streptozotocin-induced diabetic rats (Mkhwanazi et al., 2014). However, to date, there has been no report on the antifibrotic potential of MA in the kidneys.

The aim of our current study was to explore whether MA could exert anti-fibrotic effects on renal interstitial fibrosis. In addition, the role of MA in the TGF-β/Smad pathway mediated by MyD88 was also investigated.

## Materials and Methods

### Reagents and Antibodies

MA (M6699) was purchased from Sigma-Aldrich (St. Louis, MO, United States). The following primary antibodies were used for immunoblotting: anti-α-SMA (A2547; Sigma); anti-E-cadherin (#610182; BD Transduction Laboratories, San Jose, CA, United States); anti-fibronectin (SC-71114), anti-β-actin (SC-47778), anti-CTGF (SC-14939), anti-Smad6 (SC-25321), and Smad7 (SC-11392), anti-p-(NF)-κB-P65 (SC-33020), anti-(NF)-κB-P65 (SC-372) and MyD88 siRNA (SC-106986) from Santa Cruz Biotechnology (Santa Cruz, CA, United States); anti-GAPDH (AM4300; Ambion, Austin, TX, United States); anti-Lamin B antibody (ab16048, Abcam, Cambridge, United Kingdom); and anti-TGF-β (#3711S), anti-T-Smad2/3 (#3102S), anti-p-Smad2/3 (#8828S), anti-Smad4 (#38454S), anti-MyD88 (#4283S), and anti-vimentin (#5741S) from Cell Signaling Technology (Danvers, MA, United States); anti-F4/80 (MCA497GA, Bio-Rad, Hercules, CA).

### Animal Experiments

Five-week-old male C57BL/6 mice (15–20 g) were purchased from Samtako (Osan, South Korea). Mice were randomized into three groups: sham-operated, UUO, and MA-treated UUO (UUO + MA) groups (*n* = 6 each). UUO was generated by ligation of the left ureter. The abdominal cavity was exposed through a midventral incision, and a 40 silk ligature was placed at the left proximal ureter under isoflurane anesthesia (792632; Sigma). The sham group received the same surgical procedures without ureter ligation. After 24 h of ligation, the UUO + MA group was intraperitoneally injected with MA at 20 mg/kg/day for 6 days (dissolved in 20 µl DMSO), based on a previous report (Ampofo et al., 2019). Sham-operated group was treated with DMSO. All mice were euthanized and the kidneys were harvested on day 7. All animal experiments were approved by the Animal Care Regulations Committee of Chonnam National University Medical School (CNUHIACUC-20033), and our protocols conformed to the institutional guidelines for experimental animal care and use.

### Cell Culture and Treatment

Rat kidney interstitial fibroblast (NRK49F) cells and rat proximal tubular epithelial (NRK-52E) cells (American Type Culture Collection, Manassas, VA, United States) were cultured in high-glucose Dulbecco’s modified Eagle’s medium (DMEM; Welgene, Daegu, South Korea) containing 5% fetal bovine serum (FBS) and 1% streptomycin/penicillin at 37°C under a 5% CO_2_ atmosphere. After starvation with 0.5% FBS medium for 24 h, cells were pretreated with MA or vehicle-alone (DMSO) for 2 h and then incubated with TGF-β (10 ng/ml) for 30 min and harvested or displace to 5% FBS medium for 24 h. The starvation of NRK49F cell and NRK52E cell was incubated with LPS (25 ng/ml or 200 ng/ml) for 2 h. Recombinant TGF-β was purchased from Pepro Tech (Rocky Hill, NJ, United States). LPS was purchased from Sigma Chemical (Escherichia Coli 0111:B4, St. Louis, MO). MA was dissolved in dimethyl sulfoxide to obtain a 20 mM stock solution and stored at −20°C.

### Histology

Tissue sections were fixed with 4% paraformaldehyde and then embedded in paraffin sections. After dewaxing, the slides were processed for hematoxylin and eosin (H&E) staining. The kidney slides were stained with Gill’s hematoxylin for 5 min, washed with tap water, differentiated with 0.3% acid alcohol, and incubated in eosin and phloxine for 2 min. Finally, the sections were dehydrated and mounted.

Periodic acid-Schiff (PAS) staining was performed according to the manufacturer’s instructions. The tubular damage was evaluated as reversible or irreversible, previously described by Cohen A et al. (Cohen, 2007). The reversible damaged tubular was identified as the brush border absence. The irreversible damage was recognized by the presence of mild dilatation; flattened epithelial cells and loss of brush border; denudation of basement membranes, tubular necrosis, and cell apoptosis. The damaged tubules per histological field were averaged, obtaining 10 values per animal for each variable.

For Masson’s trichrome staining, the sections were deparaffinized and rehydrated through a series of 100, 95, and 70% alcohol and then re-fixed in Bouin’s solution for 1 h at 56°C to improve staining quality. After rinsing with running tap water for 5–10 min to remove the yellow color, the sections were incubated in Weigert’s iron hematoxylin solution and kept in Biebrich scarlet/acid fuchsin solution for 10–15 min.

The sections were differentiated in phosphotungstic-phosphomolybdic acid solution for 10 min, transferred directly to aniline blue solution for 10 min, subsequently incubated with acetic acid for 2 min, and rapidly dehydrated with ethanol and xylene. Collagen deposition, nuclei, and muscle fibers were stained blue, black, and red, respectively.

### Real-Time PCR

RNA from the kidney tissue was extracted with TRIzol RNA reagent (Invitrogen, Carlsbad, CA), and the concentration was quantified with NanoDrop™(Ultraspec 2000; Pharmacia Biotech, Cambridge, United Kingdom). cDNA was synthesized using Smart Cycler II System (Cepheid, Sunnyvale, CA). SYBR green was used for real-time PCR as dye to detect DNA. Relative levels of mRNA were determined by real-time PCR, using a Rotor-GeneTM 3000 Detector System (Corbette research, Mortlake, New South Wales, Australia). Sequences of primers are listed in Supplementary Table S1. The amount of the reagents was based on the instructions on the kit (GoTaq Master Mix, Cat. No. M7122). PCR conditions were as follows: 1) 95°C for 5 min; 2) 95°C for 20 s; 3) 58–60°C for 20 s (optimized for each primer pair); 4) 72°C for 30 s to detect SYBR Green. The Corbett Research Software was used to collect and evaluate data. The gene expression was calculated using the comparative critical threshold (Ct) values from quadruplicate measurements, with normalization to GAPDH as an internal control.

### Immunohistochemical Staining

Immunohistochemical staining was performed with primary α-SMA antibody (A2547; Sigma) and horseradish peroxidase-conjugated anti-mouse IgG secondary antibody (Dako, Glostrup, Denmark). Sirius red staining was performed with a Picro Sirius Red Stain Kit (ab150681; Connective Tissue Stain; Abcam, Cambridge, United Kingdom). Visual fields were selected randomly in digital images of each section under ×20 magnification. The stained sections were imaged with a Nikon microscope (Tokyo, Japan). Quantitative analysis of the stained sections was performed using ImageJ software.

### Cytotoxicity Assay

NRK49F cells were seeded into 96-well plates at a density of 1×10^4^ cells/mL in a volume of 100 μl/well and allowed to attach for 24 h. The cells were starved with 0.5% FBS medium for 24 h and then treated with the indicated amounts of MA at 10, 20, 40, 80, and 100 μM or vehicle-alone (DMSO) for an additional 24 h. Cell viability was determined by an EZ-Cytox1000 kit (Dogen, Daejeon, South Korea) following the manufacturer’s instructions. Absorbance was measured at 450 nm using a microplate reader (Bio-Tek Instruments, Winooski, VT, United States).

### siRNA Knockdown

RNA interference of MyD88 was performed using rat siRNA from Santa Cruz Biotechnology (SC-106986). Cells were transfected with either scrambled siRNA (siScr) or siRNA against MyD88 (siMyD88) at 20 nM using DharmaFECT 1 transfection reagent according to the manufacturer’s protocol (Park et al., 2019).

### Western Blotting

Total proteins from kidneys or cells frozen in liquid nitrogen were lysed in ice-cold RIPA buffer (Thermo Scientific, Waltham, MA, United States). After brief centrifugation at 13000 ×g, tissue/cell debris was removed, and the supernatant was collected. The protein concentration was determined using a BCA Protein Assay kit (Thermo Scientific) according to the manufacturer’s instructions. Equal amounts of lysates were electrophoresed using sodium dodecyl sulfate-polyacrylamide gel electrophoresis (Invitrogen, Carlsbad, CA, United States). Separated proteins were transferred to polyvinylidene fluoride membranes and blotted with specific antibodies (Kim et al., 2019). The densitometric analysis was conducted using ImageJ software.

### Nuclear Extract Preparation

To prepare nuclear extracts, cells were lysed using NE-PER nuclear extraction reagent (NER; Pierce Biotechnology, Rockford, IL, United States) according to the manufacturer’s protocol. Briefly, NRK49F cells were harvested by scraping in cold PBS and then centrifuged at 13000 ×g for 2 min. After removing the supernatant, 100 µl of ice-cold cytoplasmic extraction reagent (CER) was added to the dried cell pellets. After incubation in ice for 10 min, ice-cold CER II (5.5 µl) was added to the tube, which was centrifuged at 16000 ×g for 5 min, and the pellet fraction was suspended in 50 µl of ice-cold NER. After the tube was centrifuged at 16000 ×g for 10 min, the supernatant (nuclear extract) fraction was transferred to a new tube, and protein concentrations were measured by BCA assay.

### Statistical Analysis

The differences between groups were determined using one-way analysis of variance (ANOVA) and followed by Tukey’s post-hoc test for parametric data and Kruskal-Wallis test with Dunn’s multiple comparison post-hoc tests for nonparametric data of tubular damage. Parametric variables were expressed as mean ± SD, and nonparametric variables were expressed as medians with interquartile (25th and 75th percentiles) ranges for continuous variables. *p*-Values < 0.05 were considered statistically significant. All statistical analyses were performed using GraphPad Prism 9 (GraphPad Software, San Diego, CA).

## Results

### MA Ameliorates Histopathological Damages and Renal Fibrosis in UUO Model

**FIGURE 1:**
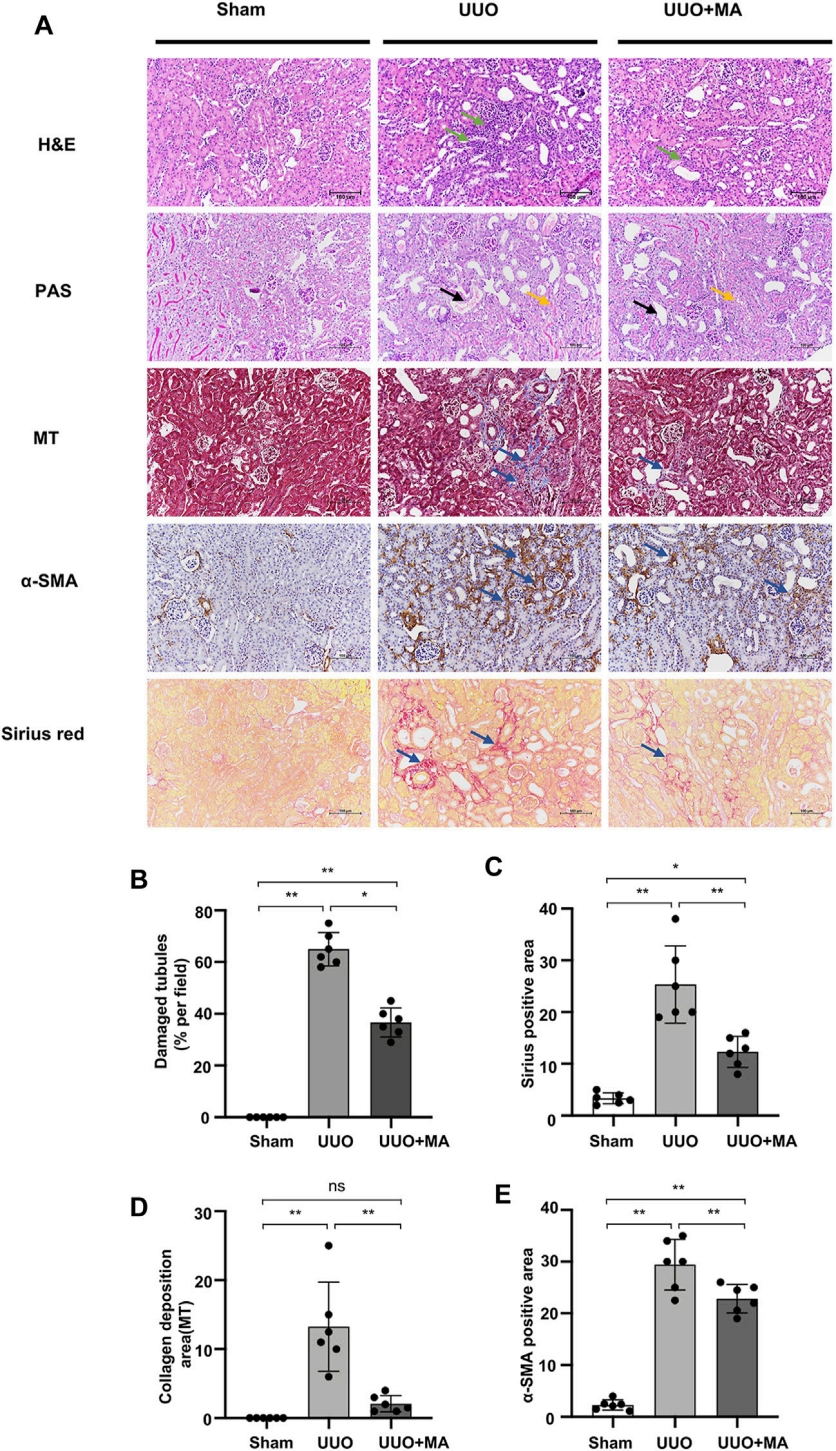
MA ameliorates histopathological damages and renal fibrosis in UUO model. **(A)** Histological changes were assessed by H&E and PAS staining. Typical interstitial tubular positive changes were indicated as follows; inflammatory cell infiltration (green arrow) in H&E stain; tubular brush border fracture (yellow arrow), and tubular atrophy and dilatation (black arrow) in PAS stain. Interstitial fibrosis (blue arrow) was assessed by MT, immunohistochemistry of α-SMA and Sirius red staining. Original magnification = ×20. Bar = 100 μm. **(B)** Kruskal-Wallis test for tubular damage, n = 6. *p < 0.05, **p < 0.01, ns: no significance. **(C–E)** Statistical significance was presented as the mean ± SD, *n* = 6. *p < 0.05, **p < 0.01, ns: no significance. HE, hematoxylin and eosin; PAS, Periodic Acid-Schiff stain; MT, Masson’s trichrome stain; MA, maslinic acid; UUO, unilateral ureteral obstruction.

We examined the effects of MA on renal interstitial fibrosis in the UUO mouse model (Figure 1 A-E. H&E staining exhibited inflammatory cell infiltration in the obstructed kidney of the UUO group, whereas a normal renal cortex was observed in the Sham group. Compared with that in the UUO group, the renal injury in the UUO + MA group was reduced. PAS staining showed tubular brush border fracture, tubular atrophy and dilatation in the UUO group. However, the UUO + MA group presented less damage in a similar area. Consistently, based on Masson’s trichrome staining, remarkable ECM deposition was evident in kidneys in the UUO group, whereas those in the UUO + MA group displayed less collagen deposition with less scope and extent of ECM deposition.

To evaluate ECM protein expression among the groups, α-SMA expression was analyzed via immunohistochemistry and collagen I/III deposition was examined using Sirius red staining. UUO kidneys showed increased immunolabeling of collagen I/III and α-SMA, which was attenuated in the UUO + MA group.

### MA Inhibits Renal Tubular ECM Deposition and EMT in UUO Model

**FIGURE 2:**
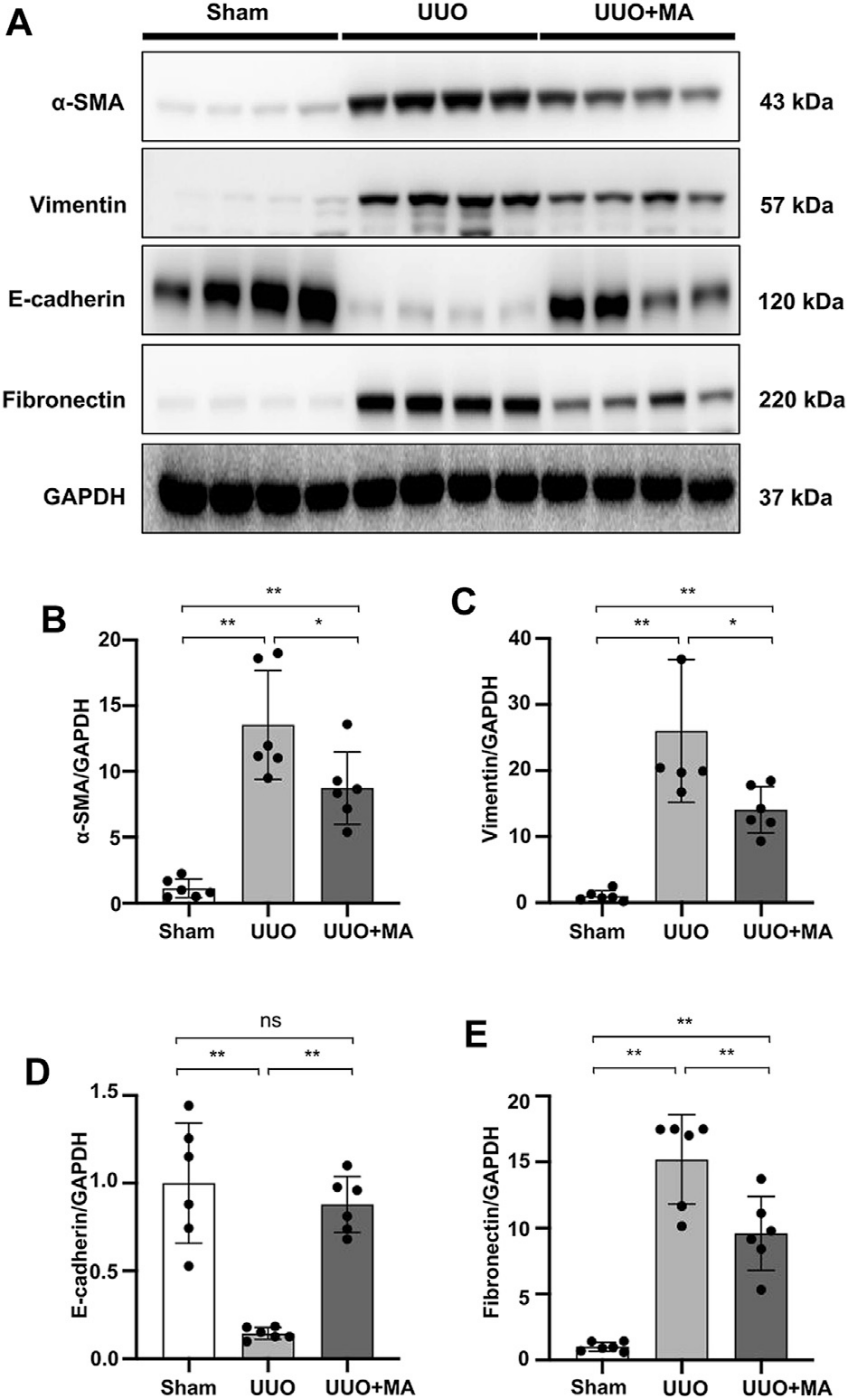
MA inhibits renal tubular ECM deposition and EMT in the UUO model. **(A)** Expression of α-SMA, vimentin, E-cadherin, and fibronectin in kidney tissues was detected by western blot and quantified by densitometry. **(B–E)** Statistical significance was presented as the mean ± SD, *n* = 6. *p < 0.05, **p < 0.01, ns: no significance. MA, maslinic acid; UUO, unilateral ureteral obstruction.

Immunoblotting analysis revealed that MA treatment downregulated protein expression of α-SMA, vimentin, and fibronectin in kidneys by 38, 44 and 40% respectively, as compared with untreated UUO group. In contrast, E-cadherin expression was markedly downregulated in UUO kidneys, which was recovered in the UUO + MA group, by approximately 70%. (Figures 2A–E).

### MA Attenuates TGF-β-Stimulated Fibrotic Changes in NRK49F Cells

**FIGURE 3:**
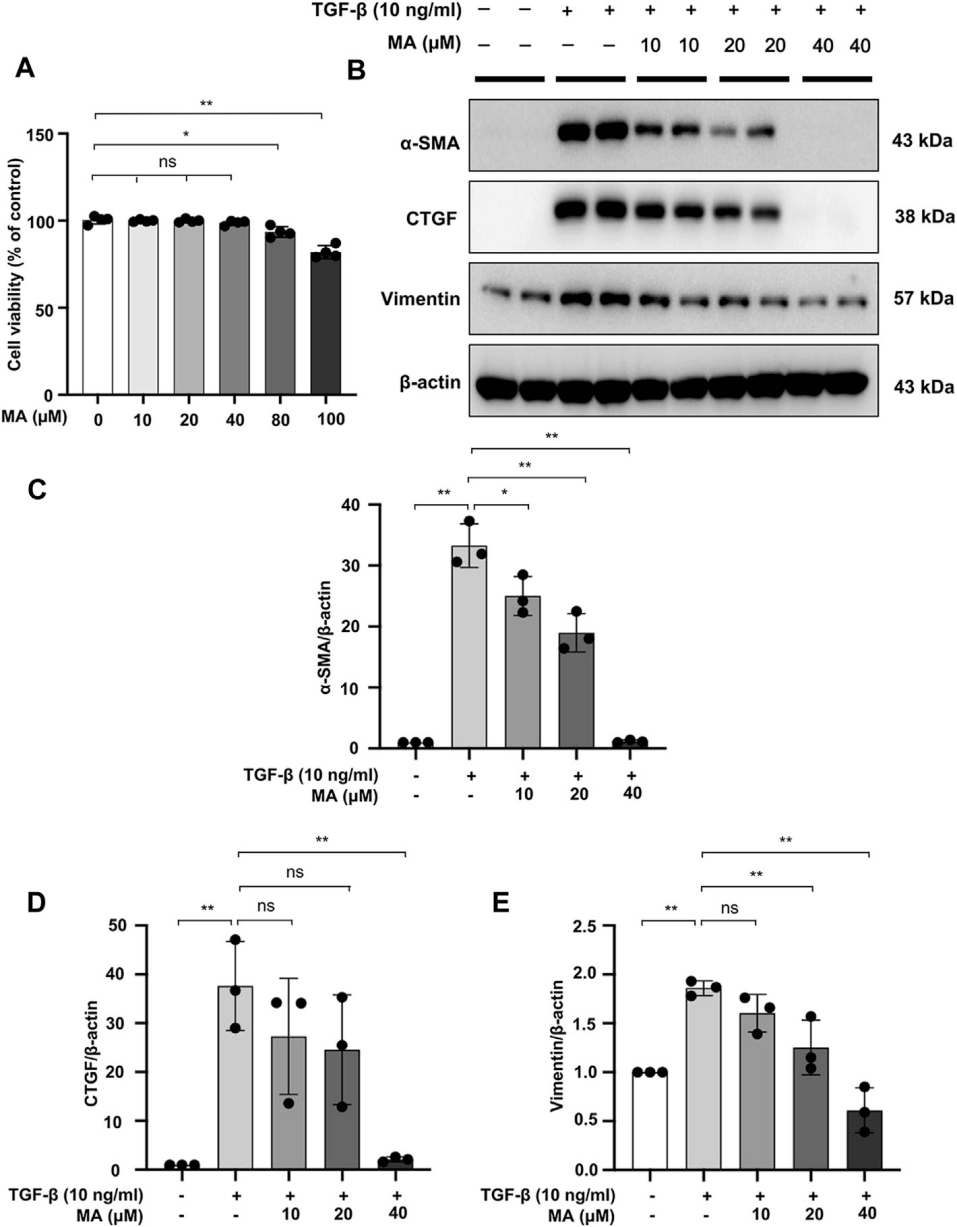
MA attenuates TGF-β-stimulated fibrotic changes in NRK49F cells. **(A)** Cells were treated with MA for 24 h, and cytotoxicity assays were conducted. **(B)** Expression of α-SMA, CTGF, and vimentin in TGF-β-stimulated NRK49F cells was detected by western blot and quantified by densitometry. Cells were pretreated with MA for 2 h after starvation with 0.5% FBS medium, treated with TGF-β (10 ng/ml) for 30 min, and incubated in 5% FBS medium for 24 h. **(C–E)** The Statistical significance was presented as the mean ± SD, *n* = 3. *p < 0.05, **p < 0.01, ns: no significance. MA, maslinic acid.

We further investigated the cytotoxic effects of MA on TGF-β-stimulated NRK49F cells (Figure 3A). Cytotoxicity assay showed no statistically significant changes in cell viability following treatment with 10–40 μM of MA, suggesting that the MA concentration in this range had no cytotoxic effect on NRK49F cells. TGF-β induced expression level of α-SMA, CTGF, and vimentin by TGF-β treatment was gradually reduced in a dose-dependent manner with the pretreatment of MA (40 μM MA treatment decreased to 10, 7.8 and 38% of TGF-β induced cell levels) (Figures 3B–E).

### MA Suppresses the TGF-β/Smad Signaling Pathway and Downregulates MyD88 Expression in UUO Model and NRK49F Cells

**FIGURE 4:**
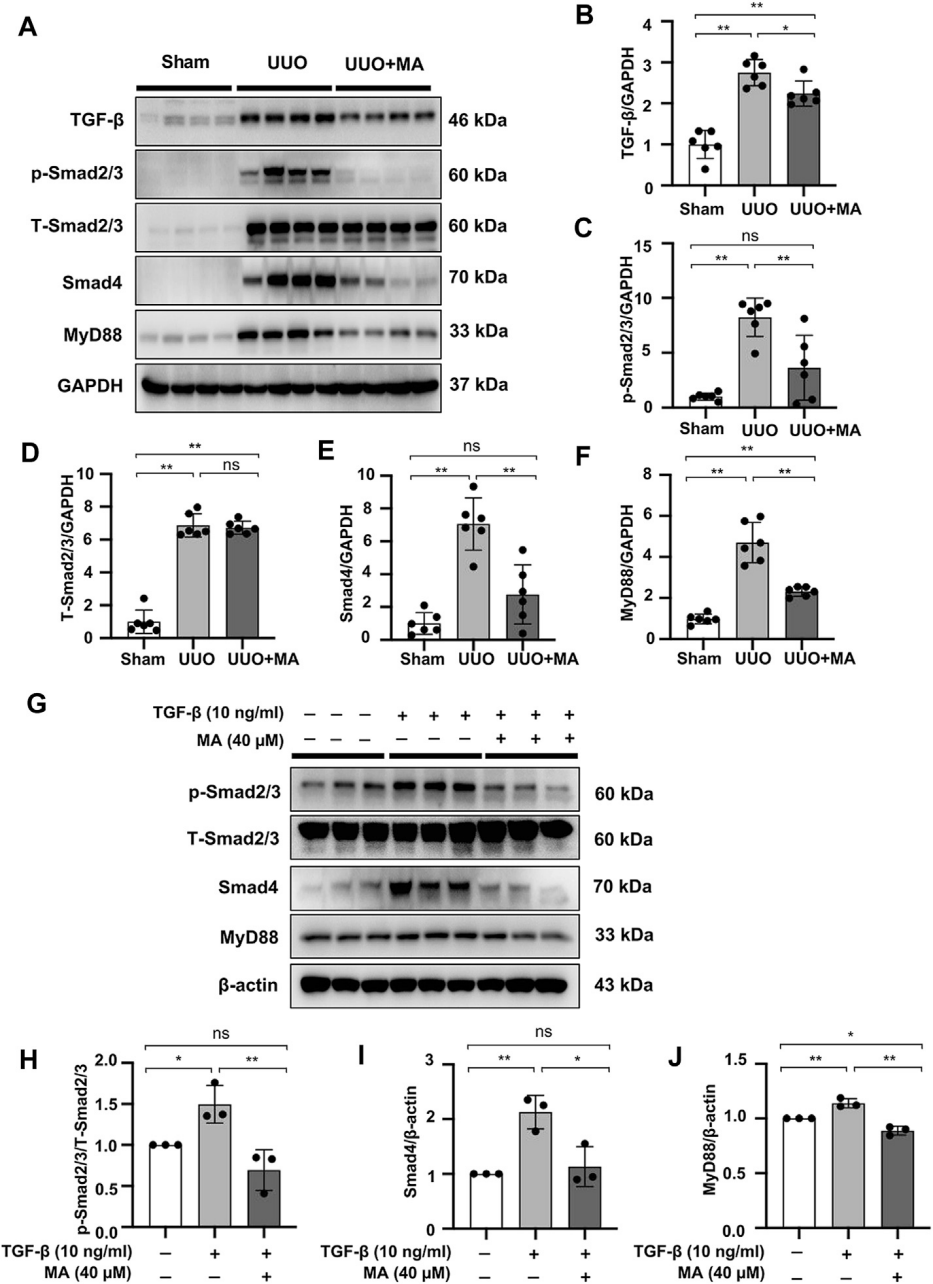
MA suppresses the TGF-β/Smad signaling pathway and downregulates MyD88 expression in UUO model and NRK49F cells. **(A)** Expression of TGF-β, p-Smad2/3, T-Smad2/3, Smad4, and MyD88 in the kidney model was detected by western blot and quantified by densitometry. **(B–F)** Statistical significance was presented as the mean ± SD, *n* = 6. *p < 0.05, **p < 0.01, ns: no significance. **(G)** Expression of p-Smad2/3, T-Smad2/3, Smad4, and MyD88 in TGF-β-treated NRK49F cells was detected by western blot and quantified by densitometry. Cells were pretreated with MA for 2 h after starvation with 0.5% FBS medium and then treated with TGF-β (10 ng/ml) for 30 min **(H–J)** Statistical significance was presented as the mean ± SD, *n* = 3. *p < 0.05, **p < 0.01, ns: no significance. MA, maslinic acid; UUO, unilateral ureteral obstruction; RT-PCR, Real-Time PCR.

As mentioned above, the TGF-β/Smad signaling pathway plays a key role in the pathogenesis of kidney fibrosis. UUO kidneys exhibited upregulated expression of TGF-β, p-Smad2/3, and Smad4. However, MA treatment significantly suppressed the expression of these fibrotic makers in UUO kidneys (Figures 4A–E). We further demonstrated that TGF-β-induced Smad2/3 phosphorylation and Smad4 expression were attenuated by pretreating NRK49F cells with MA (Figures 4G–I).

Also, the expression of MyD88 in the UUO model was significantly upregulated compared with that in the sham group, whereas its expression was remarkably downregulated following MA treatment (Figures 4A,F). Moreover, MyD88 expression induced after TGF-β stimulation was downregulated by MA pretreatment (Figures 4G,J). MyD88 mRNA levels were increased in UUO kidneys and TGF-β treated NRK49F cells. These increases were also reversed by MA treatment (Supplementary Figures S1A,B).

### MA Reduces the TGF-β-Induced Nuclear Localization of Smad2/3 and Smad4 in TGF-β-Stimulated NRK49F Cells

**FIGURE 5:**
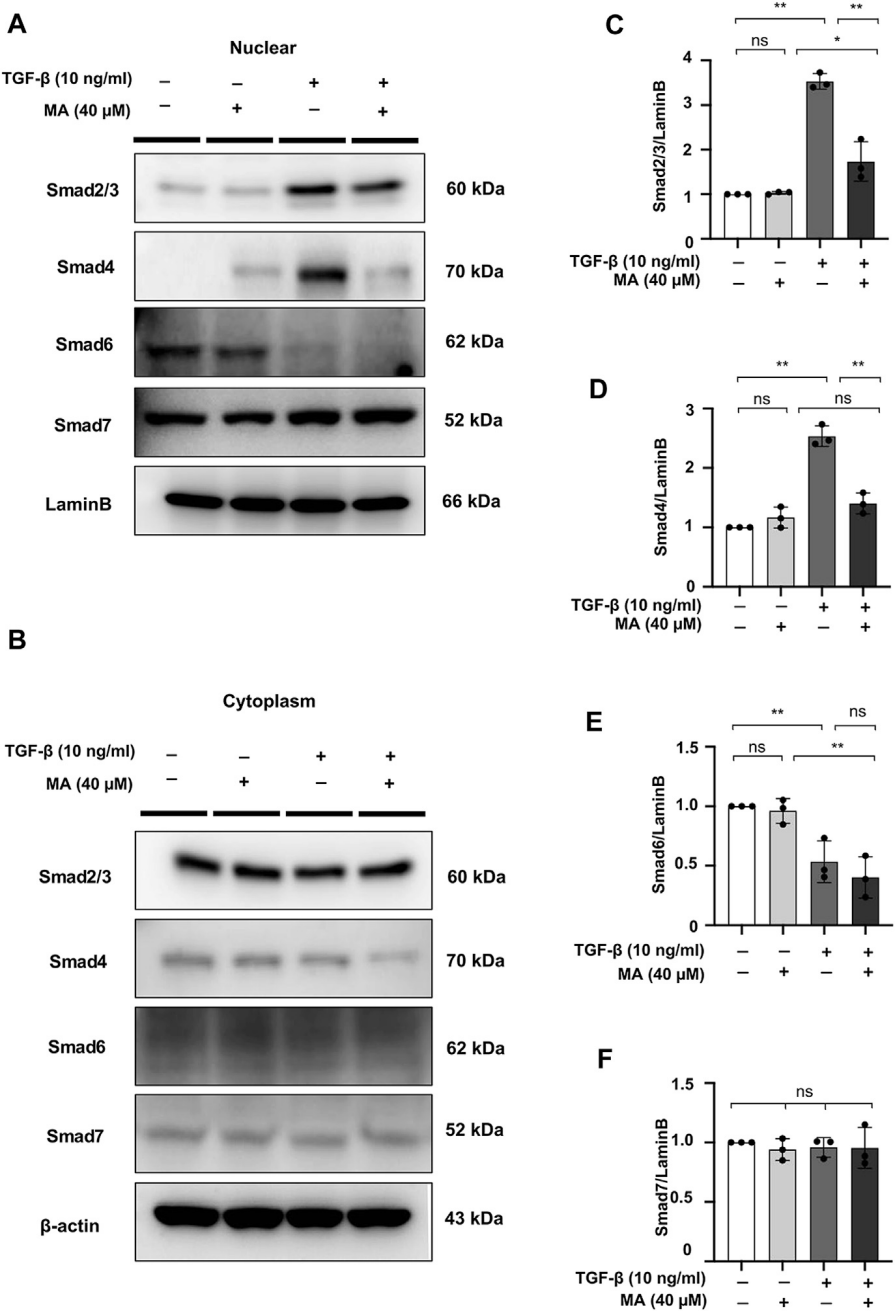
MA reduces the TGF-β-induced nuclear localization of Smad2/3 and Smad4 in TGF-β-stimulated NRK49F cells. **(A,B)** Western blotting revealed that Smad2/3 and Smad4 nuclear translocation was blocked by MA, MA does not affect Smad6 or Smad7 nuclear expression in TGF-β-stimulated NRK49F cells. NRK49F cells were pre-treated with MA for 2 h and then treated with TGF-β (10 ng/ml) for 30 min for Smad2/3, Smad4 and Smad7 or 24 h for Smad6. Cytoplasmic and nuclear proteins were separated using CER/NER buffer. β-actin and Lamin B indicate the cytoplasmic and nuclear fractions, respectively. **(C–F)** Statistical significance was presented as the mean ± SD, *n* = 3. *p < 0.05, **p < 0.01, ns: no significance.

The effect of MA on Smad signaling in response to TGF-β was further examined by Smad nuclear translocation. Pre-incubating NRK49F cells with MA significantly reduced the TGF-β–induced Smad2/3 and Smad4 nuclear translocation, but did not affect the nuclear expression of Smad6 and Smad7 (Figures 5A–F).

### MyD88 Silencing Inhibits Fibrosis in TGF-β-Stimulated NRK49F Cells

**FIGURE 6:**
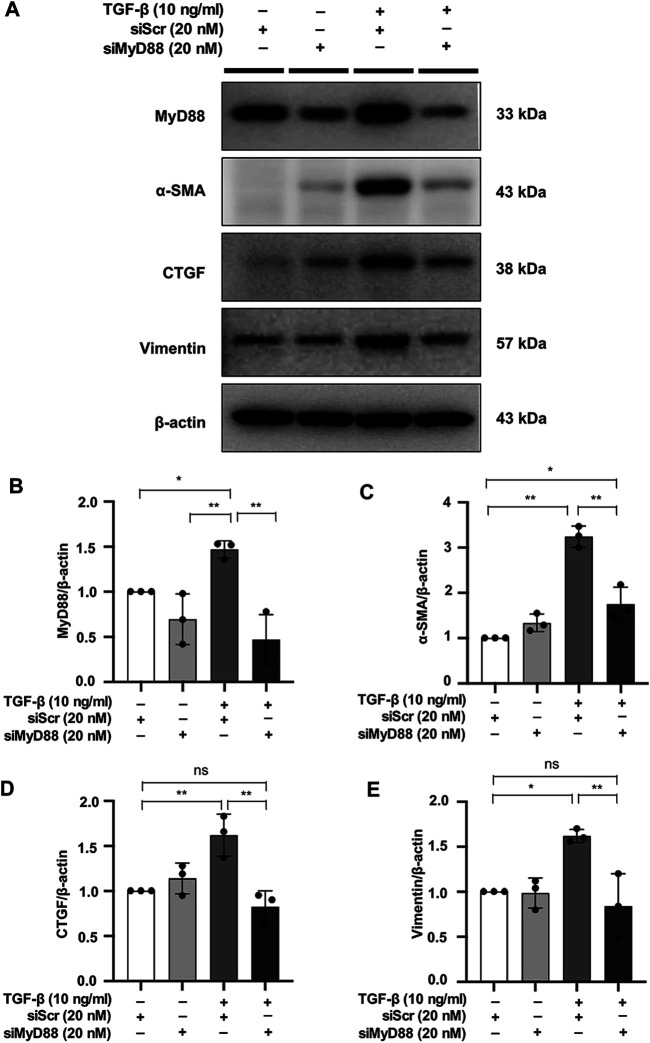
MyD88 silencing inhibits fibrosis in TGF-β-stimulated NRK49F cells. **(A)** Western blotting demonstrated that MyD88 silencing facilitated the effect on the protein expression of α-SMA, CTGF, and vimentin in TGF-β-stimulated NRK49F cells. NRK49F cells were transfected with siMyD88 or siScr for 24 h in high-glucose DMEM without FBS, starved with 0.5% FBS medium, treated with TGF-β (10 ng/ml) for 30 min, and then incubated in 5% FBS medium for 24 h. Western blotting demonstrated that MyD88 expression was significantly reduced by the specific siRNA. Results were normalized to β-actin expression. **(B–E)** Statistical significance was presented as the mean ± SD, *n* = 3. *p < 0.05, **p < 0.01, ns: no significance. MA, maslinic acid; siRNA, small interfering RNA.

To evaluate the close relationship between MyD88 and fibrotic changes in NRK49F cells, cells were transfected with siRNA against MyD88 and then subjected to TGF-β insult for 30 min. After 24 h in fresh media, TGF-β-induced increased expression level of α-SMA, CTGF, and vimentin was significantly reduced by MyD88 knockdown (Figures 6A–E).

### MyD88 Silencing Hinders Nuclear Expression of Smad4 in TGF-β-Stimulated NRK49F Cells

**FIGURE 7:**
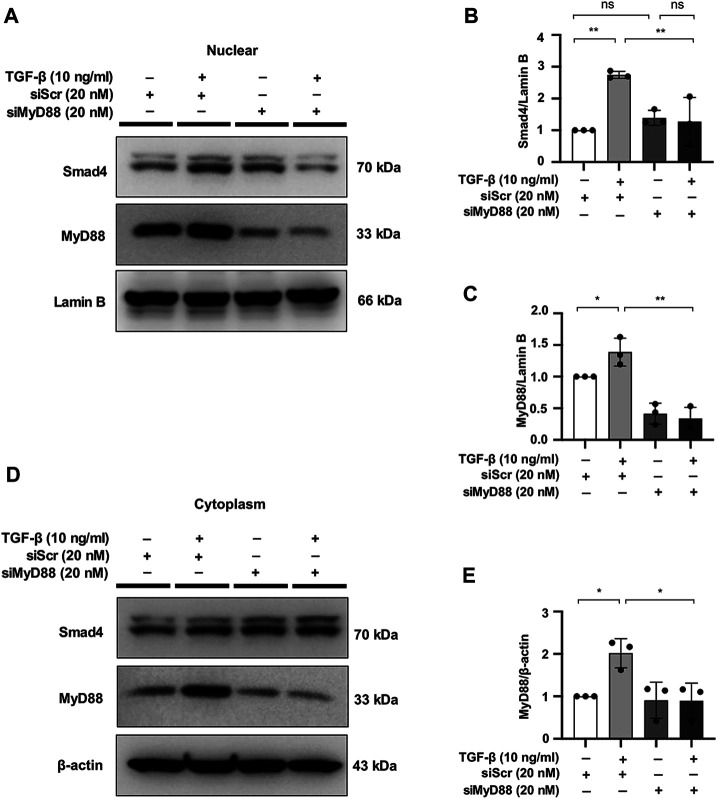
MyD88 silencing hinders nuclear expression of Smad4 in TGF-β-stimulated NRK49F cells. **(A,D)** Western blotting revealed that Smad4 nuclear translocation was blocked by siMyD88 transfection in TGF-β-stimulated NRK49F cells. NRK49F cells were transfected with siMyD88 or siScr for 24 h in high-glucose DMEM without FBS, starved with 0.5% FBS medium, and then treated with TGF-β (10 ng/ml) for 30 min. Western blotting demonstrated that MyD88 expression was significantly reduced by the specific siRNA. Cytoplasmic and nuclear proteins were separated using CER/NER buffer. β-actin and Lamin B indicate the cytoplasmic and nuclear fractions, respectively. **(B,C,E)** Statistical significance was presented as the mean ± SD, *n* = 3. *p < 0.05, **p < 0.01, ns: no significance. MA, maslinic acid; siRNA, small interfering RNA.

In a previous study, MyD88 was shown to be directly upstream of the Smad4 signaling cascade (Samba-Mondonga et al., 2018). To evaluate the effect of MyD88 on the TGF-β/Smad signaling pathway, we examined the effect of MyD88 knockdown on Smad4 expression in NRK49F cells. Nuclear expression of Smad4 was induced by TGF-β within 30 min, which was significantly downregulated in the nucleus after transfection of siRNA targeting MyD88 (Figures 7A–E).

### MA Ameliorates Renal Macrophage Infiltration and Inflammation in UUO Mice Model Associated With the Suppression of NF-κB Signaling

**FIGURE 8:**
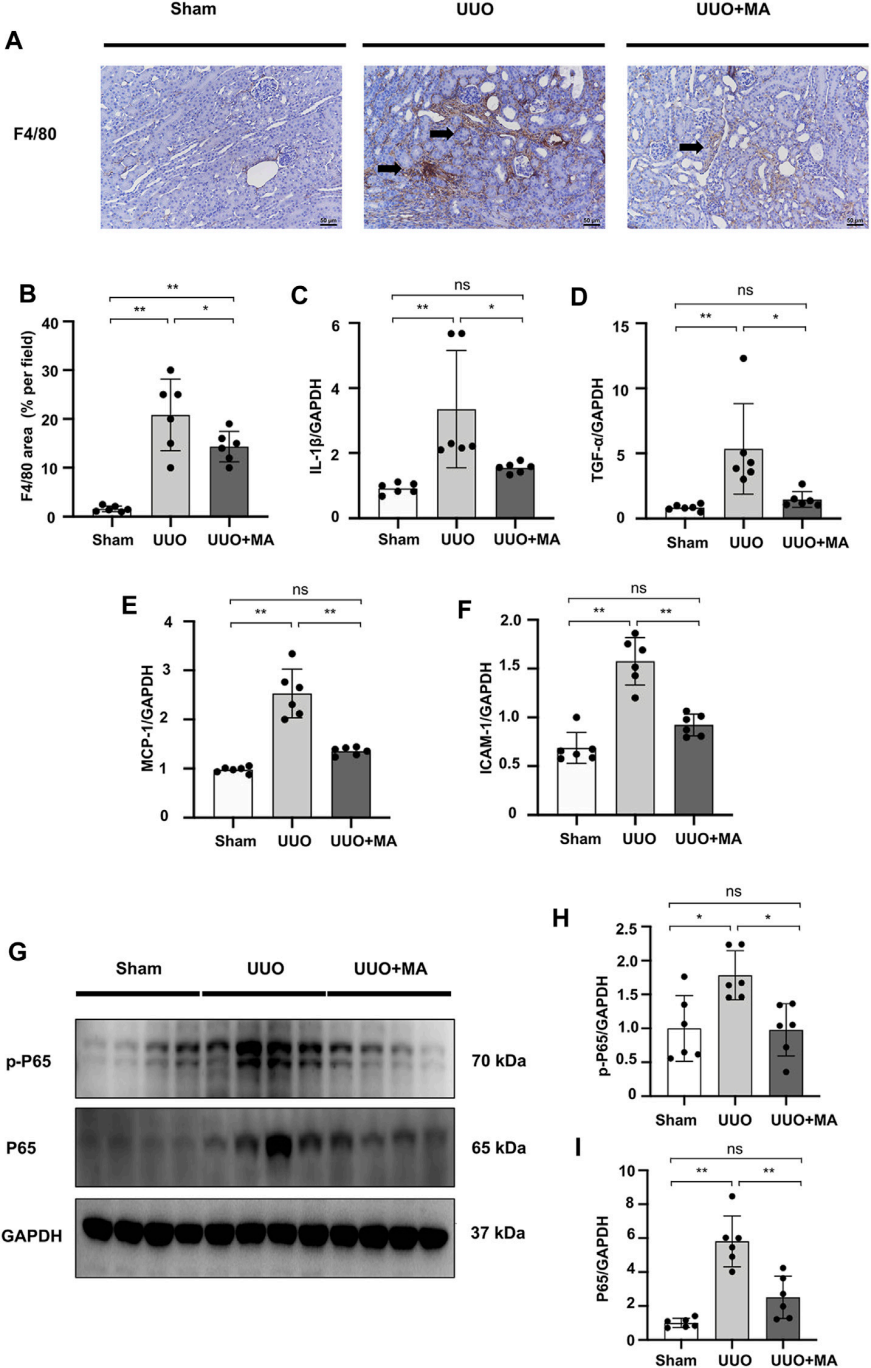
MA ameliorates renal macrophage infiltration and inflammation in UUO mice model associated with the suppression of NF-κB signaling. **(A)** Expression of F4/80 in kidneys was detected by immunohistochemistry with positive areas indicated by black arrows. Original magnification = ×20. Bar = 50 μm. **(C–F)** mRNA expression levels of IL-1β, TNF-α, MCP-1 and ICAM-1 were evaluated in UUO mice detected by RT-PCR. Results were normalized to GAPDH expression. Statistical significance was presented as the mean ± SD, *n* = 6. *p < 0.05, **p < 0.01, ns: no significance. **(G)** The protein levels of p-P65, P65 were detected by western blot and quantified by densitometry. (B,H,I) Statistical significance was presented as the mean ± SD, *n* = 6. *p < 0.05, **p < 0.01, ns: no significance. MA, maslinic acid; UUO, unilateral ureteral obstruction; RT-PCR, Real-Time PCR.

As the F4/80 molecule was established as a unique marker of murine macrophages, we assessed the effects of MA on the production of macrophages marker F4/80. Immunohistochemistry showed that there was clearly positive F4/80 antigen expression in UUO kidneys, while it was significantly reduced by MA treatment (Figures 8A,B).

We evaluated the mRNA levels of pro-inflammatory cytokines and cell adhesion molecules. Real-time PCR revealed that the mRNA levels of IL-1β, TNF-α, MCP-1, and ICAM-1 were increased in UUO kidneys, which was reversed by MA pretreatment (Figures 8C–F). We further investigated the underlying signaling mechanisms by which MA protects against inflammation. To elucidate NF-κB signaling in UUO kidneys, we detected the protein level of p-P65 and P65. Compared with sham group, the protein levels of total p-P65 and P65 were significantly increased in UUO kidneys, while the MA treatment reversed these changes (Figures 8G–I).

### MA Attenuates LPS-Induced Inflammation in NRK49F Cells and Suppresses NF-κB Signaling

**FIGURE 9:**
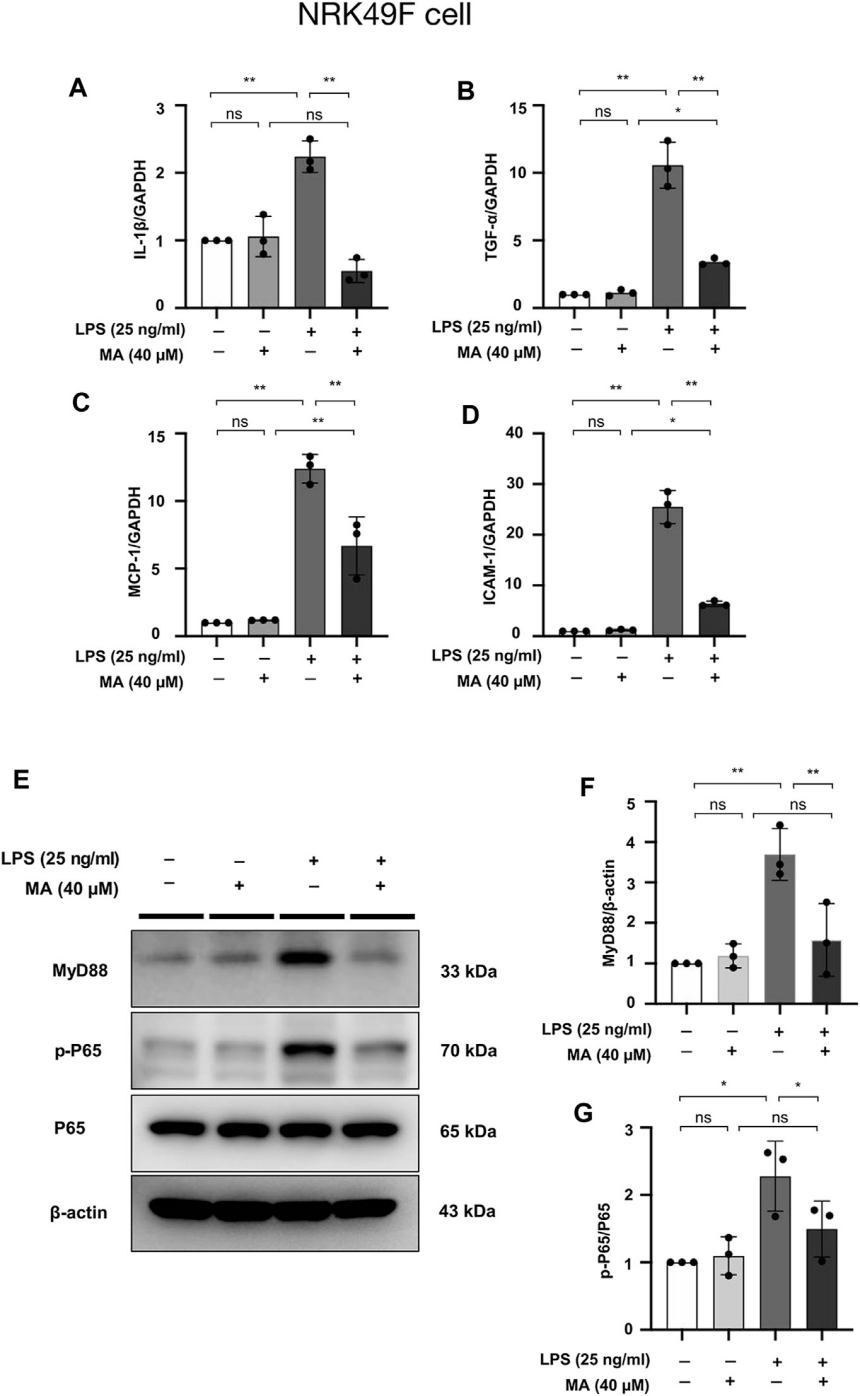
MA attenuates LPS-induced inflammation in NRK49F cells and suppresses NF-κB Signaling. **(A–D)** mRNA expression levels of IL-1β, TNF-α, MCP-1 and ICAM-1 were evaluated in LPS-treated NRK49F cells and determined by RT-PCR. Cells were pretreated with MA for 2 h after starvation with 0.5% FBS medium, then treated with LPS (25 ng/ml) for 2 h. Results were normalized to GAPDH expression. The data are presented as the mean ± SD, *n* = 3. *p < 0.05, **p < 0.01, ns: no significance. MA, maslinic acid; LPS, Lipopolysaccharide; RT-PCR, Real-Time PCR. **(E)** Expression of p-P65, P65 and MyD88 in LPS-treated NRK49F cells was detected by western blot and quantified by densitometry. Cells were pretreated with MA for 2 h after starvation with 0.5% FBS medium and then treated with LPS (25 ng/ml) for 2 h. **(F,G)** Statistical significance was presented as the mean ± SD, *n* = 3. *p < 0.05, **p < 0.01, ns: no significance. MA, maslinic acid; LPS, Lipopolysaccharide.

mRNA levels of IL-1β, TNF-α, MCP-1, and ICAM-1 were increased in NRK49F cells by LPS treatment, the changes of which were ameliorated by MA co-administration (Figures 9A–D). These results supported the notion that MA exerted anti-inflammatory effect against the UUO injury and LPS. The protein expressions of MyD88 and p-P65 in LPS treated NRK49F cells were higher than those in control group and MA only group, which were reduced by MA treatment (Figures 9E–G). Taken together, these results indicated that MA prevented the MyD88 and NF-κB signaling thereby suppressing the inflammatory response in the UUO model and LPS induced NRK49F cell lines.

### MA Attenuates LPS-Induced Inflammation in NRK52E Cells and Suppresses NF-κB Signaling

**FIGURE 10:**
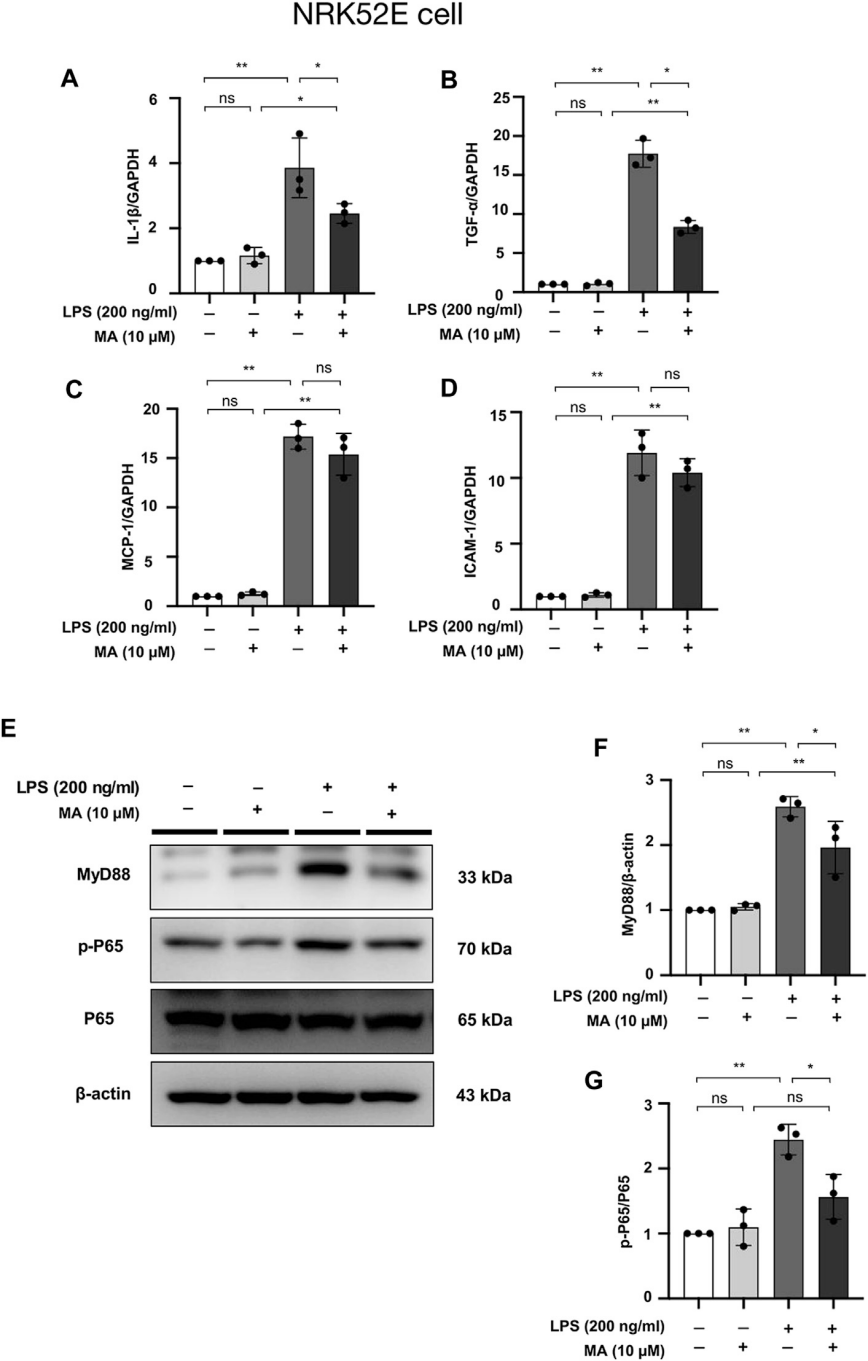
MA attenuates LPS-induced inflammation in NRK52E cells and suppresses NF-κB Signaling. **(A–D)** mRNA expression levels of IL-1β, TNF-α, MCP-1 and ICAM-1 were evaluated in LPS-treated NRK52E cells and determined by RT-PCR. Cells were pretreated with MA for 2 h after starvation with 0.5% FBS medium, then treated with LPS (200 ng/ml) for 2 h. Results were normalized to GAPDH expression. The data are presented as the mean ± SD, *n* = 3. *p < 0.05, **p < 0.01, ns: no significance. MA, maslinic acid; LPS, Lipopolysaccharide; RT-PCR, Real-Time PCR. **(E)** Expression of p-P65, P65 and MyD88 in LPS-treated NRK52E cells was detected by western blot and quantified by densitometry. Cells were pretreated with MA for 2 h after starvation with 0.5% FBS medium and then treated with LPS (200 ng/ml) for 2 h. **(F,G)** Statistical significance was presented as the mean ± SD, *n* = 3. *p < 0.05, **p < 0.01, ns: no significance. MA, maslinic acid; LPS, Lipopolysaccharide.

mRNA levels of IL-1β, TNF-α, MCP-1, and ICAM-1 checked were increased by LPS treatment in NRK52E cells, MA decreased the mRNA expressions of IL-1β and TNF-α, but not MCP-1 and ICAM-1 (Figures 10A–D). MyD88 and p-P65 protein levels were increased by LPS treatment in NRK52E cells, which were reduced in MA treatment (Figures 10E–G). We further determined whether MA also inhibits the NF-κB signaling by TGF-β stimulation. No activation of p-P65 was observed by TGF-β in NRK49F and NRK52E cells (Supplementary Figures S2A–E).

## Discussion

In this study, we found that MA plays a beneficial role to attenuate renal interstitial fibrosis by negatively regulating the TGF-β/Smad and NF-κB pathway *via* targeting MyD88. Renal interstitial fibrosis has a prominent role in the development and progression of kidney injury. It is characterized by a large accumulation of ECM, which is a complex network of collagen, elastin, several glycoproteins (e.g., fibronectin), and proteoglycans (Frantz et al., 2010; Mutsaers et al., 2015). Here, we demonstrated that MA treatment effectively downregulated ECM protein expression, suggesting that MA is effective in the treatment of renal fibrosis.

Fibroblast-to-myofibroblast transition, a representative process among several routes of exaggerated ECM accumulation in renal fibrosis, is characterized by the expression of mesenchymal cell products such as α-SMA, vimentin (intermediate filament), and collagen I (Klingberg et al., 2013; Desai et al., 2014). Additionally, downregulation of the expression and function of E-cadherin, an epithelial cell adhesion receptor essential in the maintenance of tubular epithelial integrity and cell-cell adhesion, is a phenotypic hallmark during EMT of tubular epithelial cells (Liu, 2004; Nieto et al., 2016). To investigate the effect of MA on ECM protein expression and EMT in renal fibrosis, we employed the UUO model and finally demonstrated that MA had an ameliorative effect on renal tubular injury and fibrosis. At the molecular level, we demonstrated that MA could reverse the expression of ECM, such as α-SMA, vimentin, and fibronectin. Moreover, a drastic decrease in E-cadherin in the kidney of UUO group was restored to a certain extent by MA treatment, further indicating that MA could delay EMT progression in UUO model. Collectively, the results demonstrated that MA is a beneficial effector of anti-fibrosis in the kidney of UUO model, leading to decreased ECM accumulation and EMT levels.

The increases in molecular markers, such as α-SMA, vimentin, and CTGF, represent the induction of TGF-β-stimulated fibrosis in NRK49F cells. Previous studies demonstrated that at least some pro-fibrotic effects of TGF-β were mediated through the upregulation of its downstream effector, CTGF (Biernacka et al., 2011). The downregulated expression of these fibrosis-related proteins clearly demonstrates the dose-dependent effects of MA pretreatment in NRK49F cells, which was consistent with the observation *in vivo*.

Our study also demonstrated that MA treatment attenuated the TGF-β/Smad pathway. In renal fibrosis, TGF-β is considered the master regulator of EMT and ECM accumulation (Xu et al., 2009; Bon et al., 2019). It is generally accepted that TGF-β interacts with TGF-β receptors to phosphorylate Smad2/3, subsequently activating p-Smad oligomerization with Smad4 and translocating them to the nucleus, where they jointly transactivate downstream fibrogenesis genes (Schnaper et al., 2003; Choi et al., 2012). Based on the notion that the inhibition of Smad2/3 phosphorylation will reduce fibrosis levels, we successfully identified the ability of MA to suppress Smad2/3 phosphorylation *in vivo* as well as *in vitro*, suggesting that negative regulation of MA in renal fibrosis occurs through the downregulation of p-Smad2/3, at least in part. Of interest, MA also blocked Smad2/3 and Smad4 nuclear translocation, suggesting that the inhibitory effect of MA appears to be Smad2/3 and Smad4 specific upon TGF-β stimulation. Additionally, we extend regulatory studies of MA to other Smad family members, Smad6 and Smad7 have been reported as an intracellular negative regulator of TGF-β signaling, particularly in R-Smad activation, these two factors could inhibit the phosphorylation of Smad2/3, thereby blocking TGF-β signaling (Kretzschmar and Massagué, 1998; Zimmerman and Padgett, 2000). MA treatment did not affect the nuclear expression of Smad6 and Smad7 suggesting that MA selectively downregulates nuclear activation of Smad2/3 and Smad4, and subsequently inhibits fibrosis in NRK49F cells.

The role of Myd88 in kidney fibrosis remains controversial (Skuginna et al., 2011; Anders and Lech, 2013). Recent studies suggested the abnormal activation of TLR4/Myd88 might play a role in the pathogenesis of kidney inflammation and fibrosis (González-Guerrero et al., 2017; Ma et al., 2021). In addition, Myd88 knockout mice had less UUO induced interstitial fibrosis associated with improved renal function (Braga et al., 2012). Also, inhibited activation of MyD88 could ameliorate kidney injury in UUO (Lu et al., 2018). Myd88 deficiency was protective against renal interstitial fibrosis and chronic allograft injury (Wu et al., 2012). These findings suggest that MyD88 is a potential therapeutic target for renal fibrosis. In the present study, we have demonstrated that MA treatment decreased tubulointerstitial fibrosis associated with downregulation of Myd88 and TGF-β/Smad signaling pathways. TGF-β-induced activation of α-SMA, CTGF and vimentin was reduced by Myd88 knockdown in NRK49F cells. Moreover, TGF-β induced nuclear activation of Smad4 in NRK49F cells was significantly downregulated by transfection of siRNA targeting MyD88. These findings are consistent with recent study demonstrating that MyD88 expression levels affect Smad4 protein levels in Hub7 hepatoma cells through the Toll/IL-1 receptor domain of the MyD88 protein (Samba-Mondonga et al., 2018). Taken together with the present study, MA exerts its anti-fibrotic effects on the TGF-β/Smad pathway via targeting MyD88.

The kidney damage that occurs in CKD is at least partly promoted by the immune system (Kurts et al., 2013). Different types of kidney injury and repair involve tissue remodeling mediated by the immune infiltration of renal tubular interstitial macrophages. In particular, some pan-markers like F4/80 antigen is a mature cell surface glycoprotein expressed at high levels on various macrophages (Tian et al., 2015). In this study, we detected the level of labeled F4/80 antigen. The results revealed a less infiltration of F4/80 IHC staining positive area in MA treat kidneys compared to those in UUO kidneys, which proved that MA reduces the macrophage accumulation and infiltration in UUO mice model.

Inflammation is another pathologic process engaged in renal fibrosis (Meng et al., 2014). We further examined to address the impact of MA on inflammatory state. Our results indicated that the administration of MA inhibits the expression of pro-inflammatory cytokines and cell adhesion molecules such as IL-1β, TNF-α, MCP-1, and ICAM-1, suggesting that MA may ameliorate renal fibrosis by the inhibition of inflammatory response. It has been suggested that NF-κB is a recognized downstream pro-inflammatory signaling pathway of MyD88 (Kawai and Akira, 2007), and LPS-induced NF-κB nuclear translocation is primarily dependent on MyD88 (Sakai et al., 2017). The present study also demonstrated that increased expression of p-P65 in UUO kidneys and LPS-treated cells (NRK49F cells and NRK52E cells) was counteracted by MA treatment. These changes were associated with MA induced MyD88 downregulation, and suggested that MyD88 inhibition by MA may be associated with inhibition of NF-κB signaling followed by anti-inflammatory and anti-fibrotic effects.

It should be mention that our current research has some notable limitations. Firstly, research lacks preventive design used. In addition, it remains to be elucidated whether the beneficial effect of MA is unique to the UUO kidneys or may be a more general characteristic beyond UUO in other experimental animal models involving different injury mechanisms (Yang et al., 2010). Thus, a comprehensive study on the other experimental models should be conducted over the following studies.

In conclusion, our present study suggested that MA is not only a potential agent for reducing renal fibrosis by directly targeting TGF-β/Smad signaling, but also an effective inhibitor of MyD88 that may be involved in Smad4 nuclear expression or localization and NF-κB signaling. We believe that MA may be an attractive therapeutic candidate for progressive kidney disease. Furthermore, we clarified novel cross-regulation between MyD88 and the TGF-β/Smad pathway, thus identifying MyD88 as a potential therapeutic target in renal fibrosis. These findings may contribute to the identification of novel signaling pathways involved in renal fibrosis and help to unveil the pathogenesis of CKD in the near future.

## Data Availability

The original contributions presented in the study are included in the article/Supplementary Material, further inquiries can be directed to the corresponding author.
