# Supplementary material for: Renoprotective Effects of Maslinic Acid on Experimental Renal Fibrosis in Unilateral Ureteral Obstruction Model via Targeting MyD88
Source: Front Pharmacol. 2021 Sep 13;12:708575. doi: 10.3389/fphar.2021.708575 (PMC8475766; doi:10.3389/fphar.2021.708575)
Supplement: Supplementary file 1 [file DataSheet2.PDF]

Figure.2A

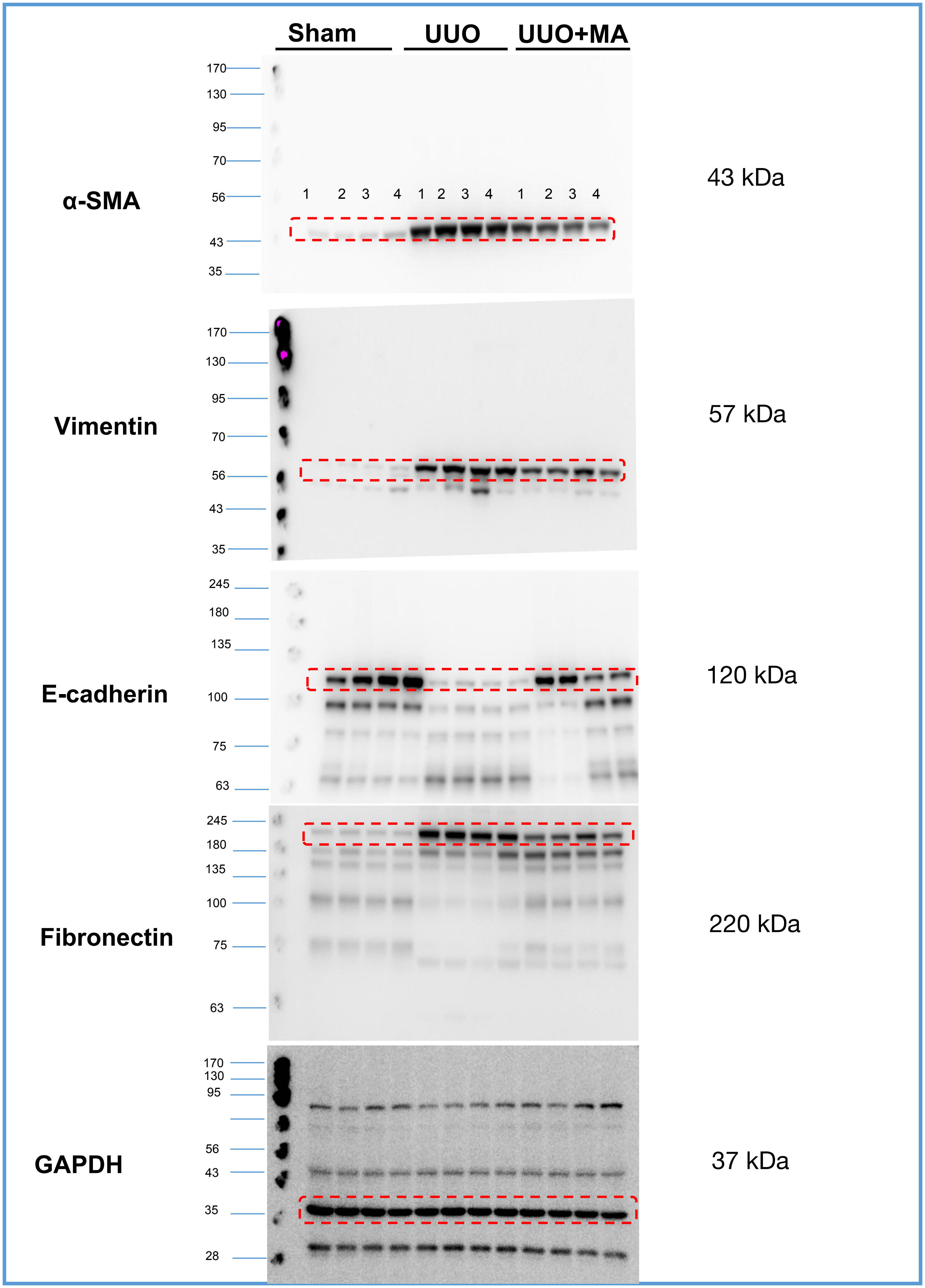

Sham
UUO
UUO+MA
α-SMA
170
130
95
70
56
43
35
1 2 3 4 1 2 3 4 1 2 3 4
43 kDa
Vimentin
170
130
95
70
56
43
35
57 kDa
E-cadherin
245
180
135
100
75
63
120 kDa
Fibronectin
245
180
135
100
75
63
220 kDa
GAPDH
170
130
95
56
43
35
28
37 kDa

Figure 3B

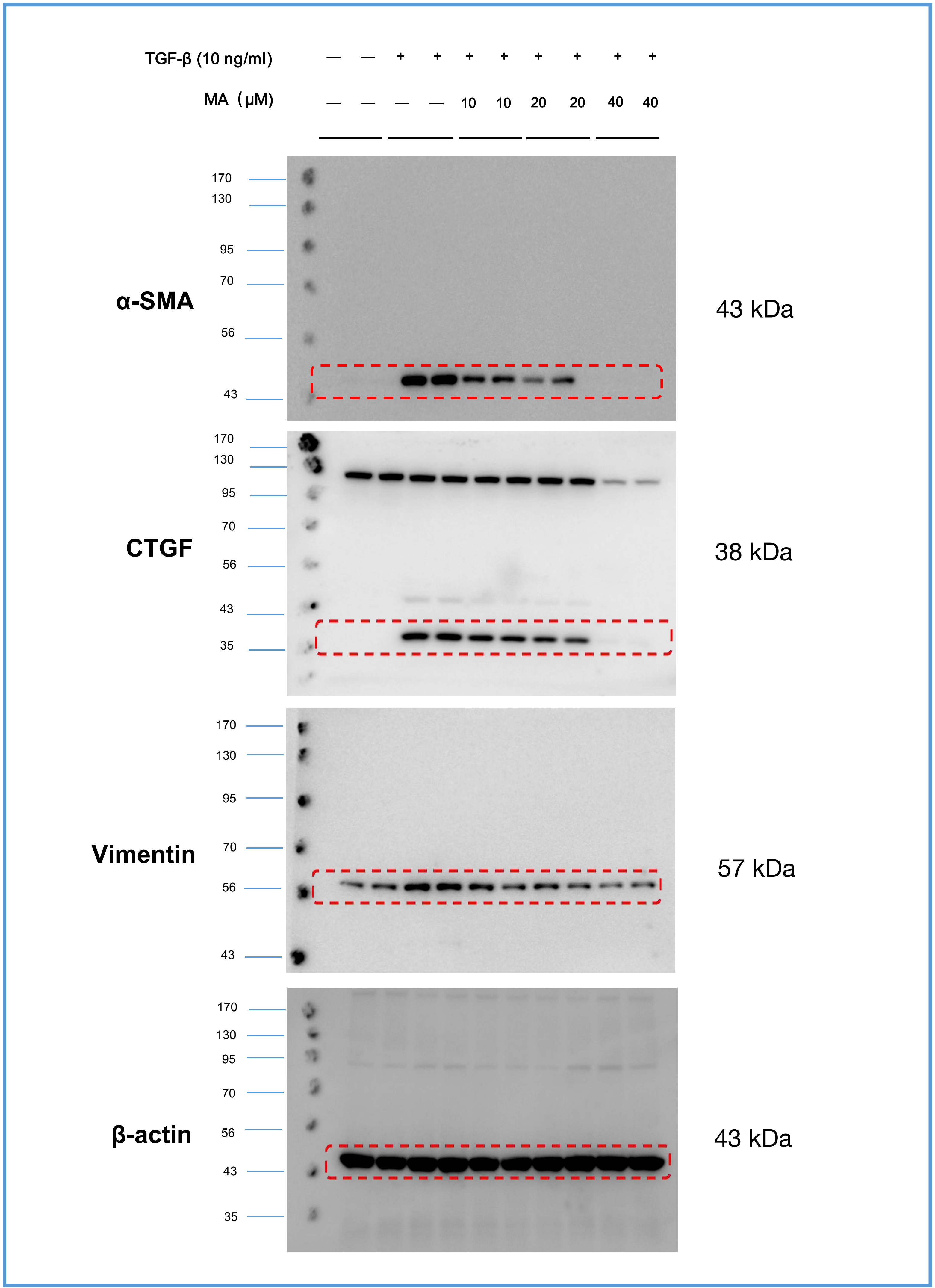
TGF-β (10 ng/ml) — — + + + + + + + +
MA（μM) — — — — 10 10 20 20 40 40
α-SMA
43 kDa
CTGF
38 kDa
Vimentin
57 kDa
β-actin
43 kDa
170
130
95
70
56
43
35

Figure 4A

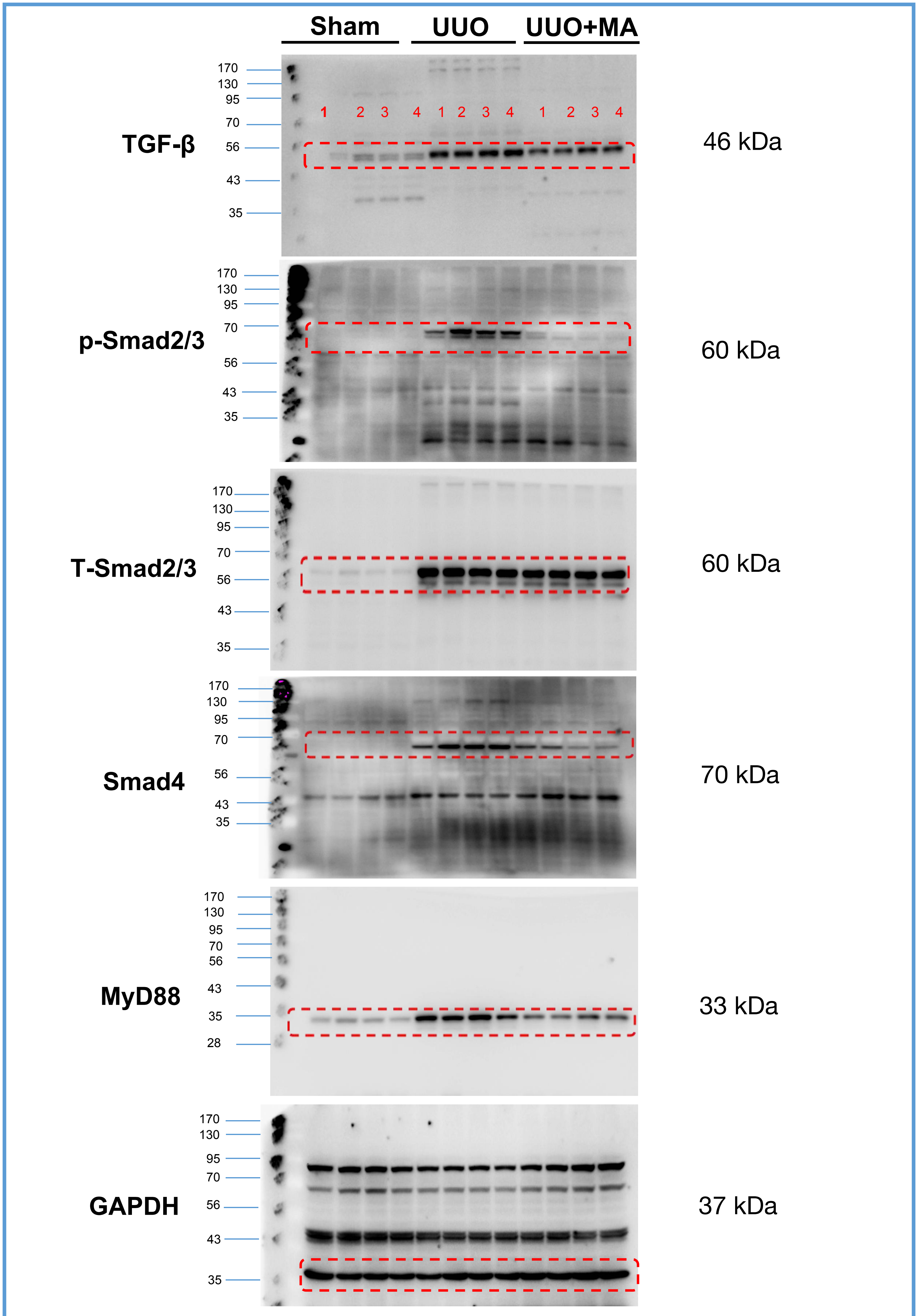

Sham
UUO
UUO+MA
TGF-β
46 kDa
p-Smad2/3
60 kDa
T-Smad2/3
60 kDa
Smad4
70 kDa
MyD88
33 kDa
GAPDH
37 kDa

Figure 4G

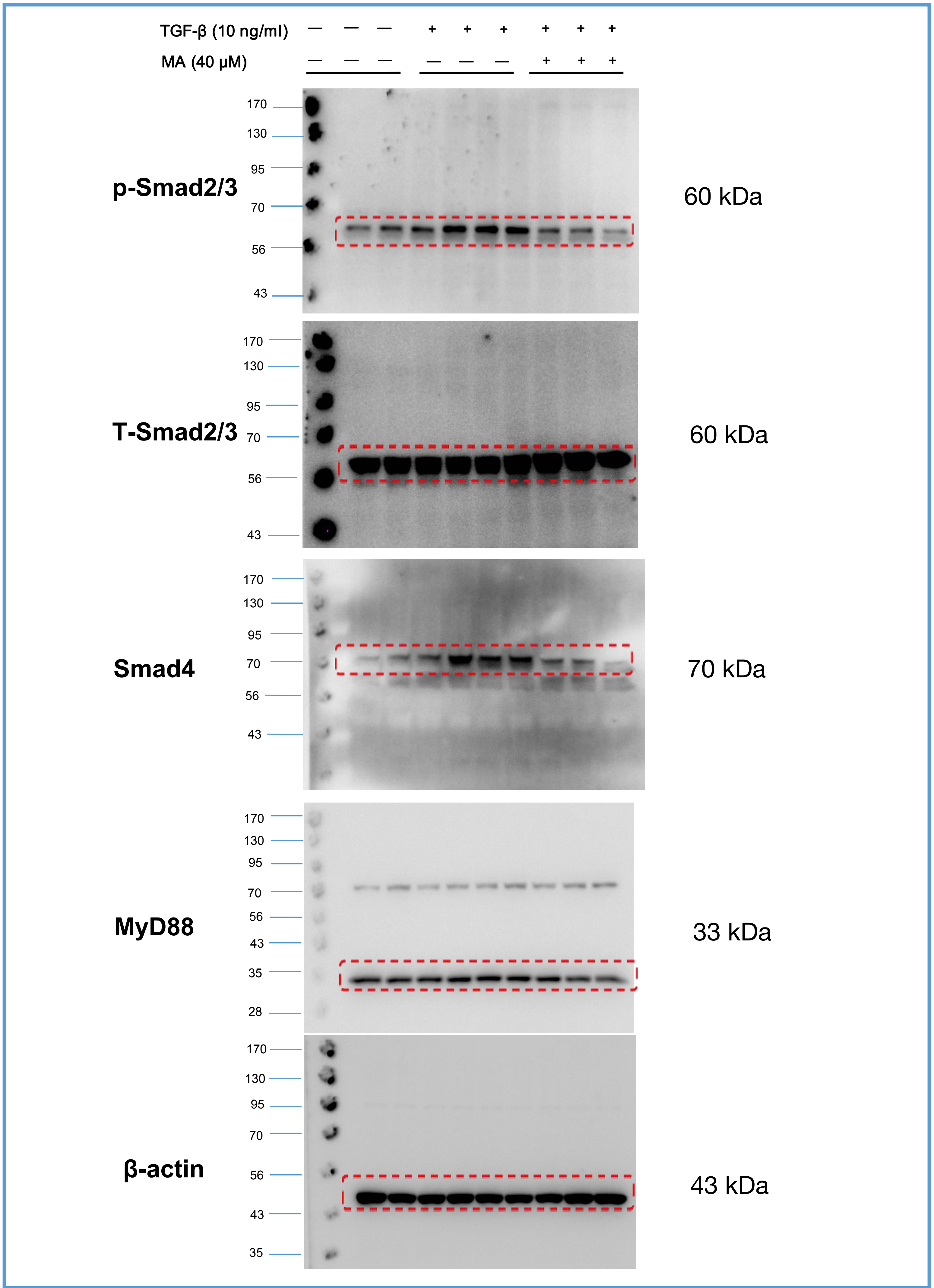

TGF-β (10 ng/ml) — — — + + + + + +
MA (40 μM) — — — — — — + + +
p-Smad2/3
60 kDa
T-Smad2/3
60 kDa
Smad4
70 kDa
MyD88
33 kDa
β-actin
43 kDa
170
130
95
70
56
43
35
28

Figure 5A

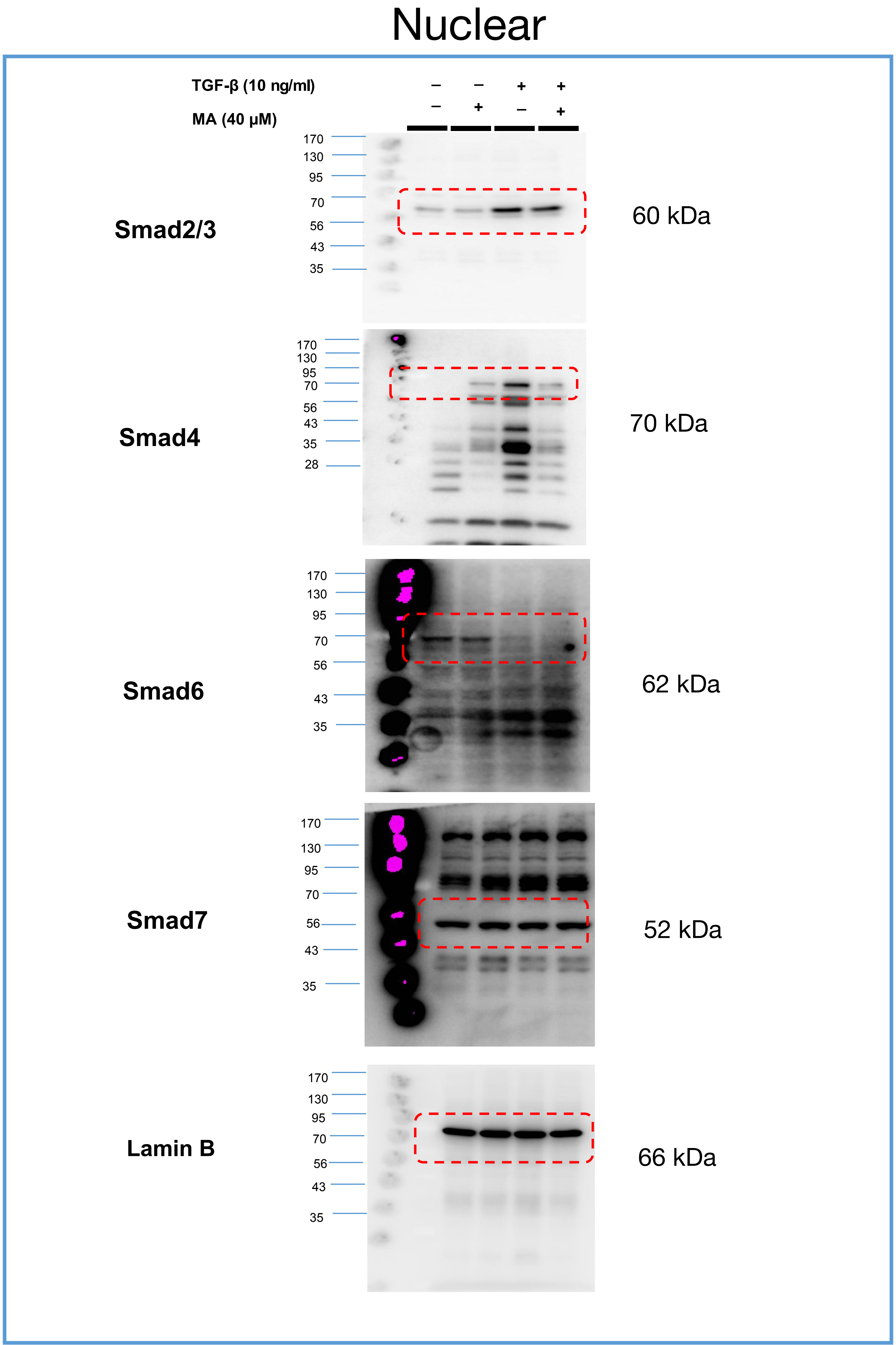
Nuclear
TGF-β (10 ng/ml)
MA (40 μM)
–
–
+
+
–
+
–
+
Smad2/3
170
130
95
70
56
43
35
60 kDa
Smad4
170
130
95
70
56
43
35
28
70 kDa
Smad6
170
130
95
70
56
43
35
62 kDa
Smad7
170
130
95
70
56
43
35
52 kDa
Lamin B
170
130
95
70
56
43
35
66 kDa

Figure 5B

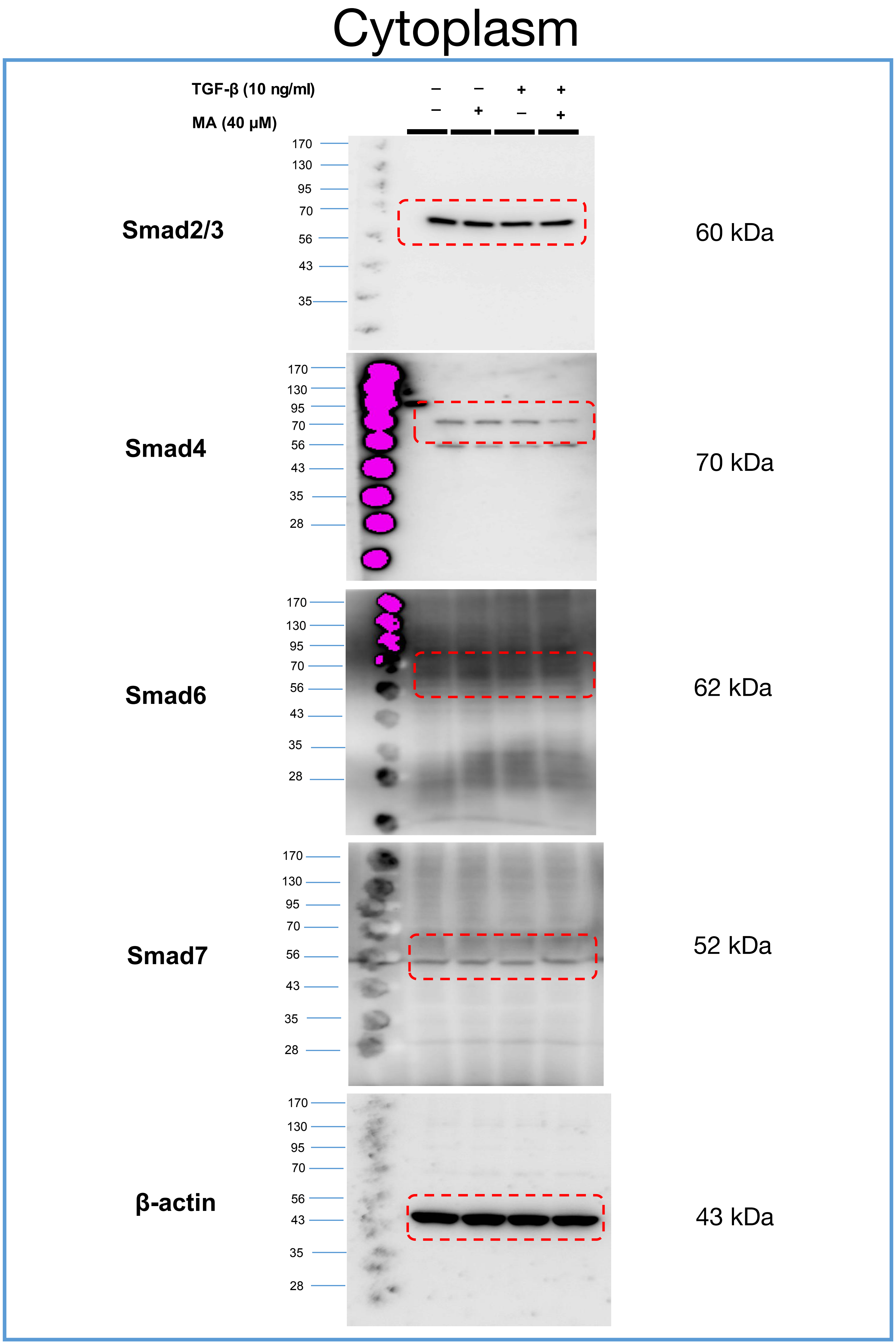

Cytoplasm
TGF-β (10 ng/ml)
MA (40 μM)
− − + +
− + − +
Smad2/3
60 kDa
Smad4
70 kDa
Smad6
62 kDa
Smad7
52 kDa
β-actin
43 kDa
170
130
95
70
56
43
35
28

Figure 6A

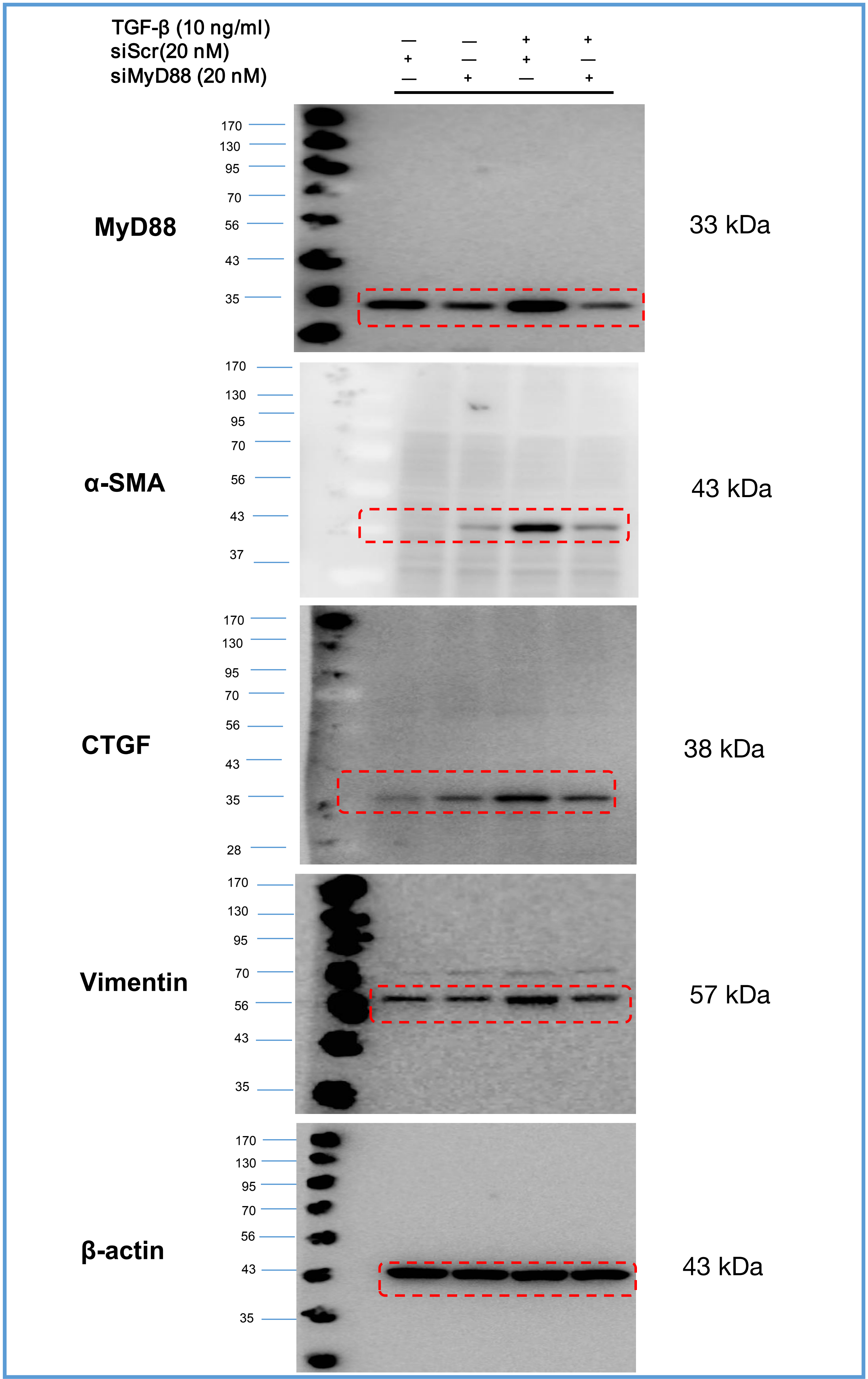

TGF-β (10 ng/ml)
siScr(20 nM)
siMyD88 (20 nM)
MyD88
α-SMA
CTGF
Vimentin
β-actin
33 kDa
43 kDa
38 kDa
57 kDa
43 kDa
170
130
95
70
56
43
37
35
28

Figure 7A

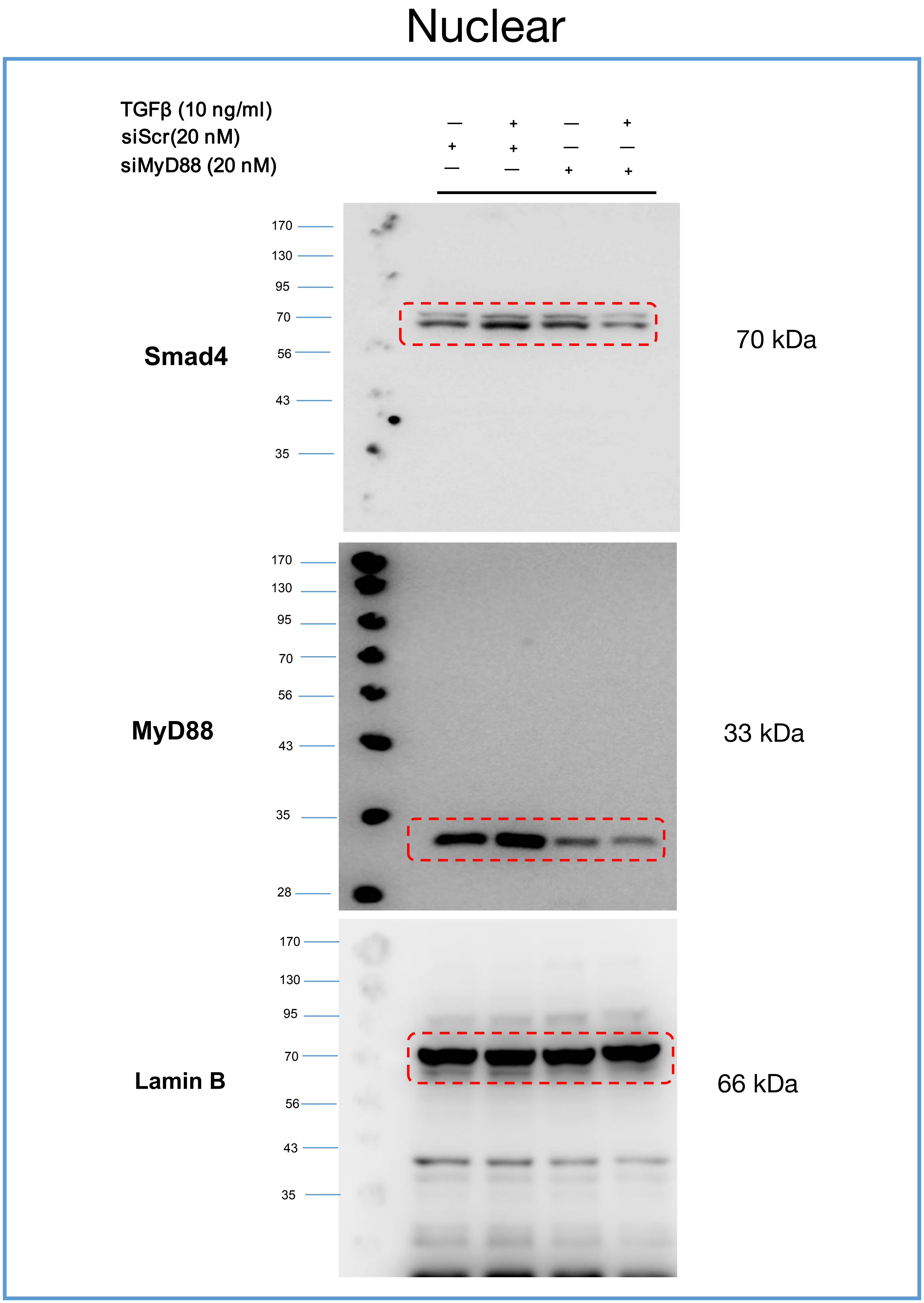
Nuclear
TGFβ (10 ng/ml)
siScr(20 nM)
siMyD88 (20 nM)
— + — +
+ + — —
— — + +
Smad4
170
130
95
70
56
43
35
70 kDa
MyD88
170
130
95
70
56
43
35
28
33 kDa
Lamin B
170
130
95
70
56
43
35
66 kDa

Figure 7D

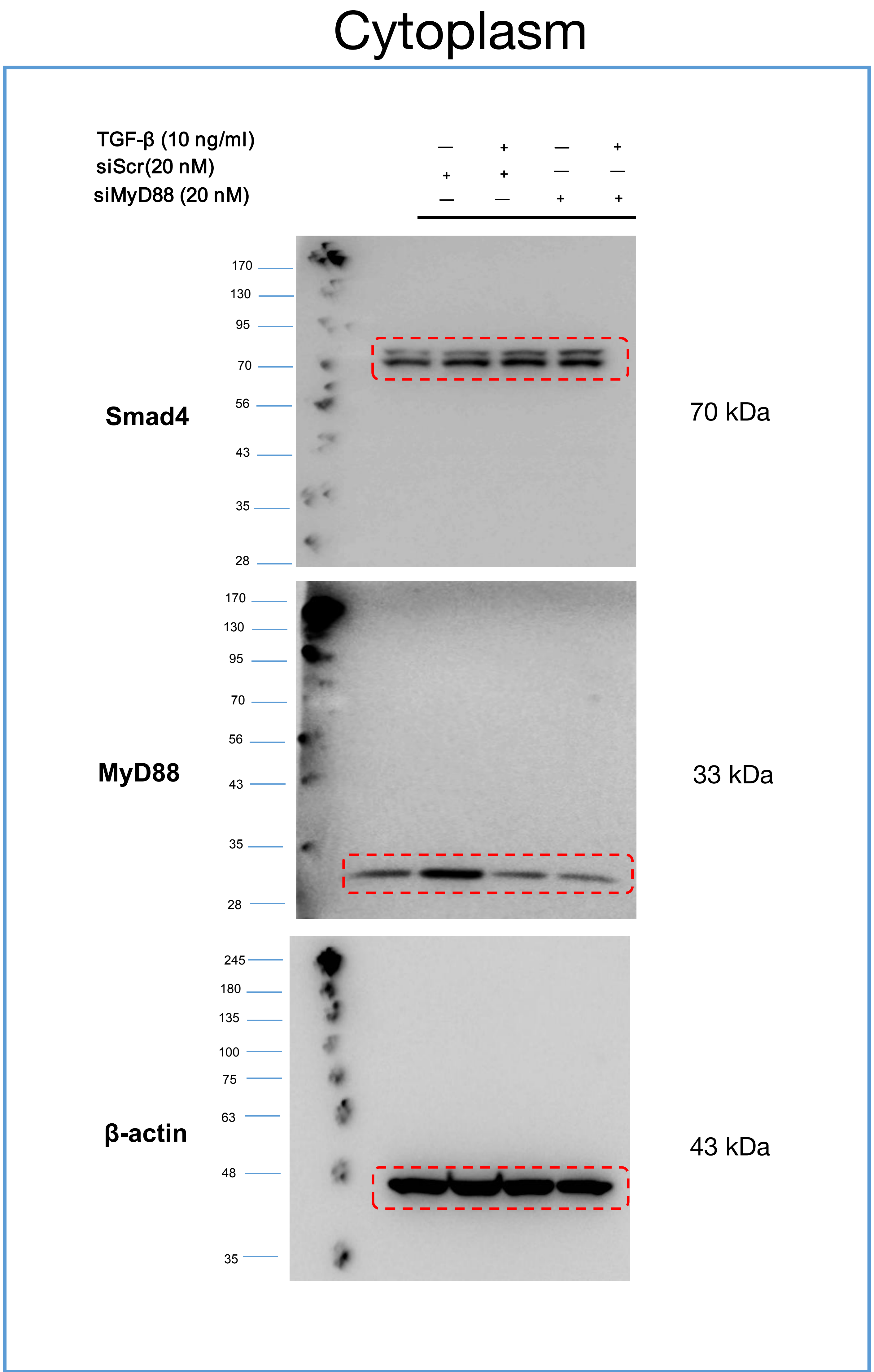
Cytoplasm
TGF-β (10 ng/ml)
siScr(20 nM)
siMyD88 (20 nM)
— + — +
+ + — —
— — + +
Smad4
170
130
95
70
56
43
35
28
70 kDa
MyD88
170
130
95
70
56
43
35
28
33 kDa
β-actin
245
180
135
100
75
63
48
35
43 kDa

Figure 8E

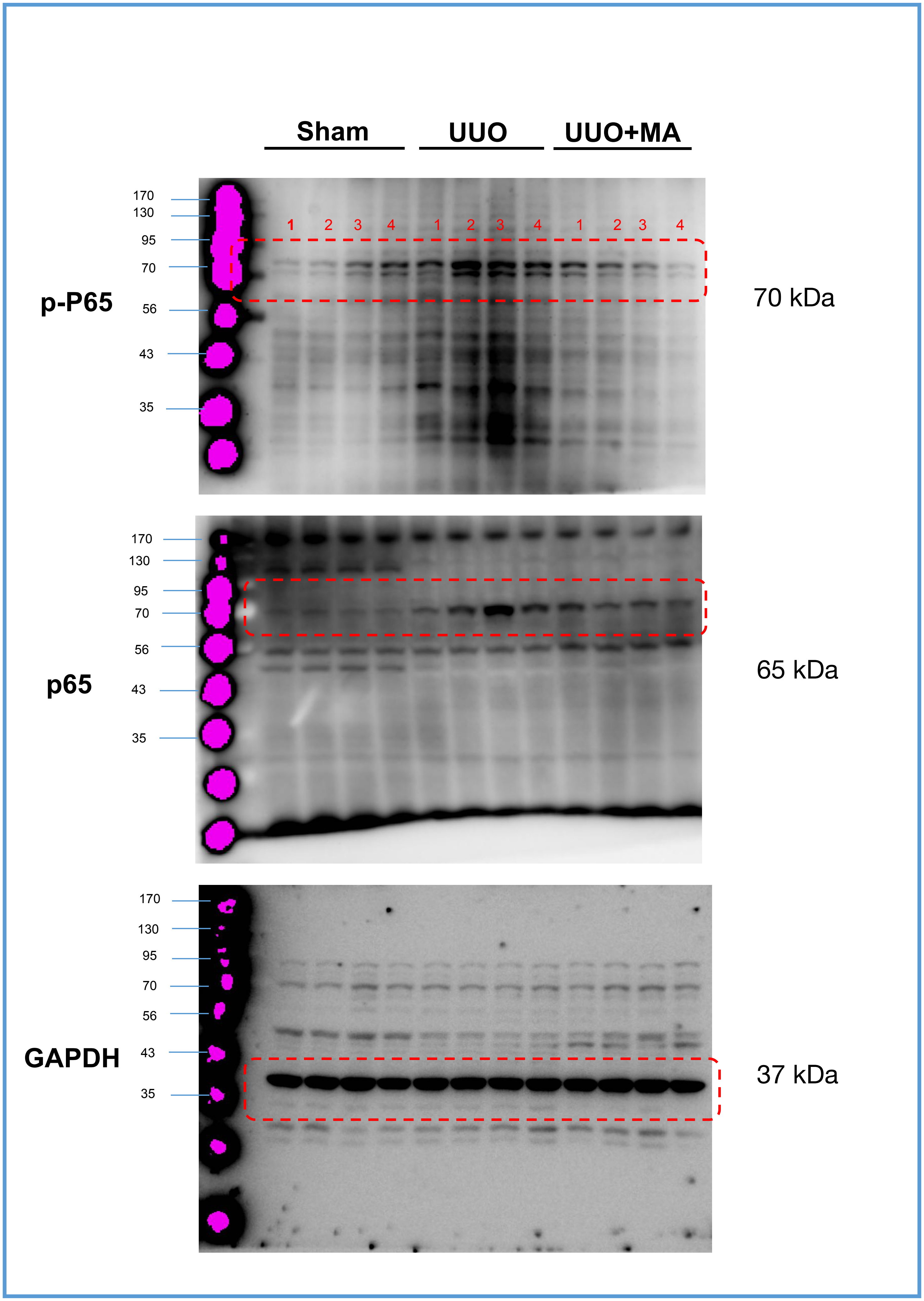

Sham
UUO
UUO+MA
p-P65
170
130
95
70
56
43
35
1 2 3 4 1 2 3 4 1 2 3 4
70 kDa
p65
170
130
95
70
56
43
35
65 kDa
GAPDH
170
130
95
70
56
43
35
37 kDa

Figure 9E

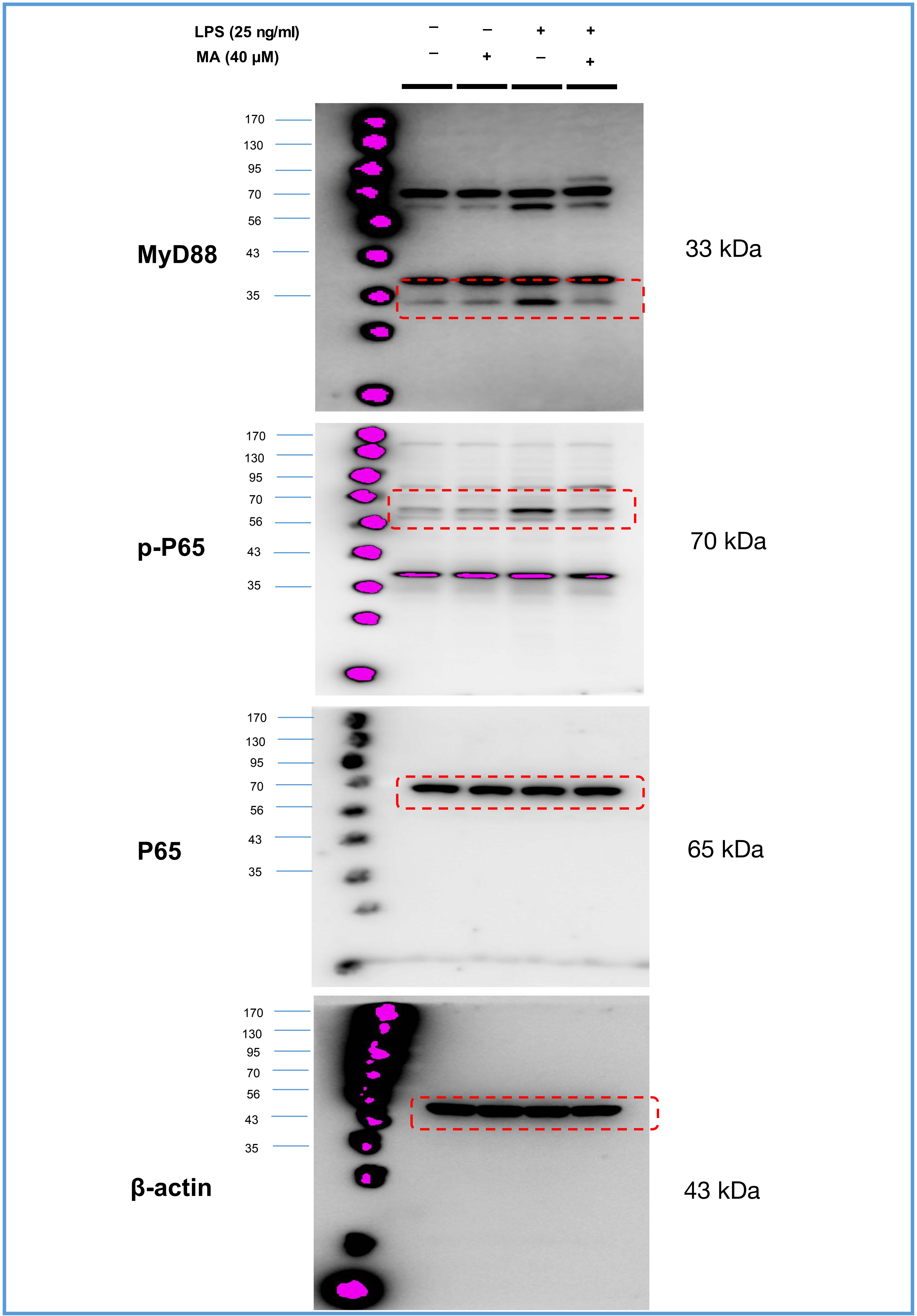
LPS (25 ng/ml) – – + +
MA (40 μM) – + – +
170
130
95
70
56
43
35
MyD88
33 kDa
p-P65
70 kDa
P65
65 kDa
β-actin
43 kDa

Figure 10E

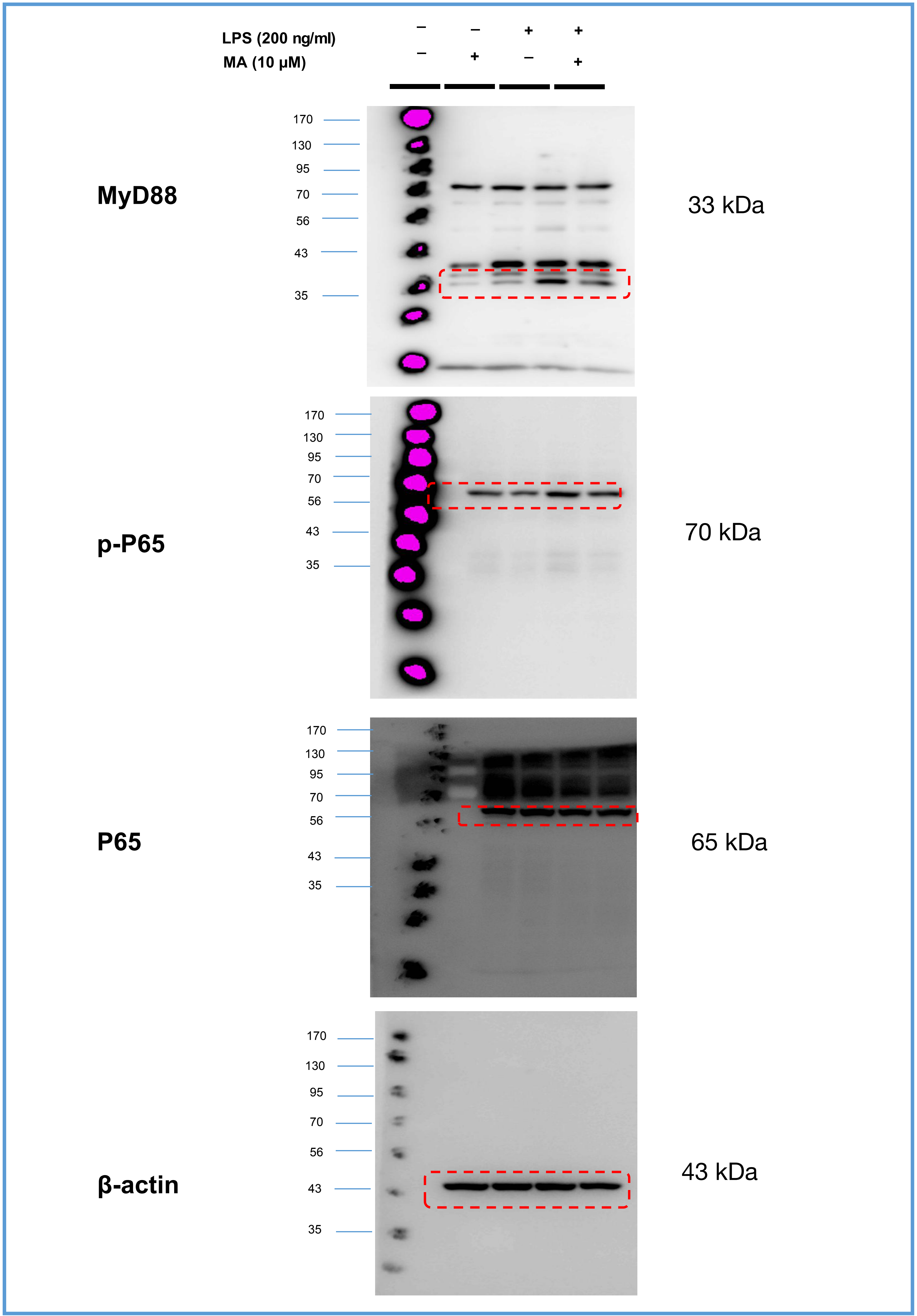

LPS (200 ng/ml) − − + +
MA (10 μM) − + − +
MyD88
33 kDa
p-P65
70 kDa
P65
65 kDa
β-actin
43 kDa
170
130
95
70
56
43
35

Figure S2 A

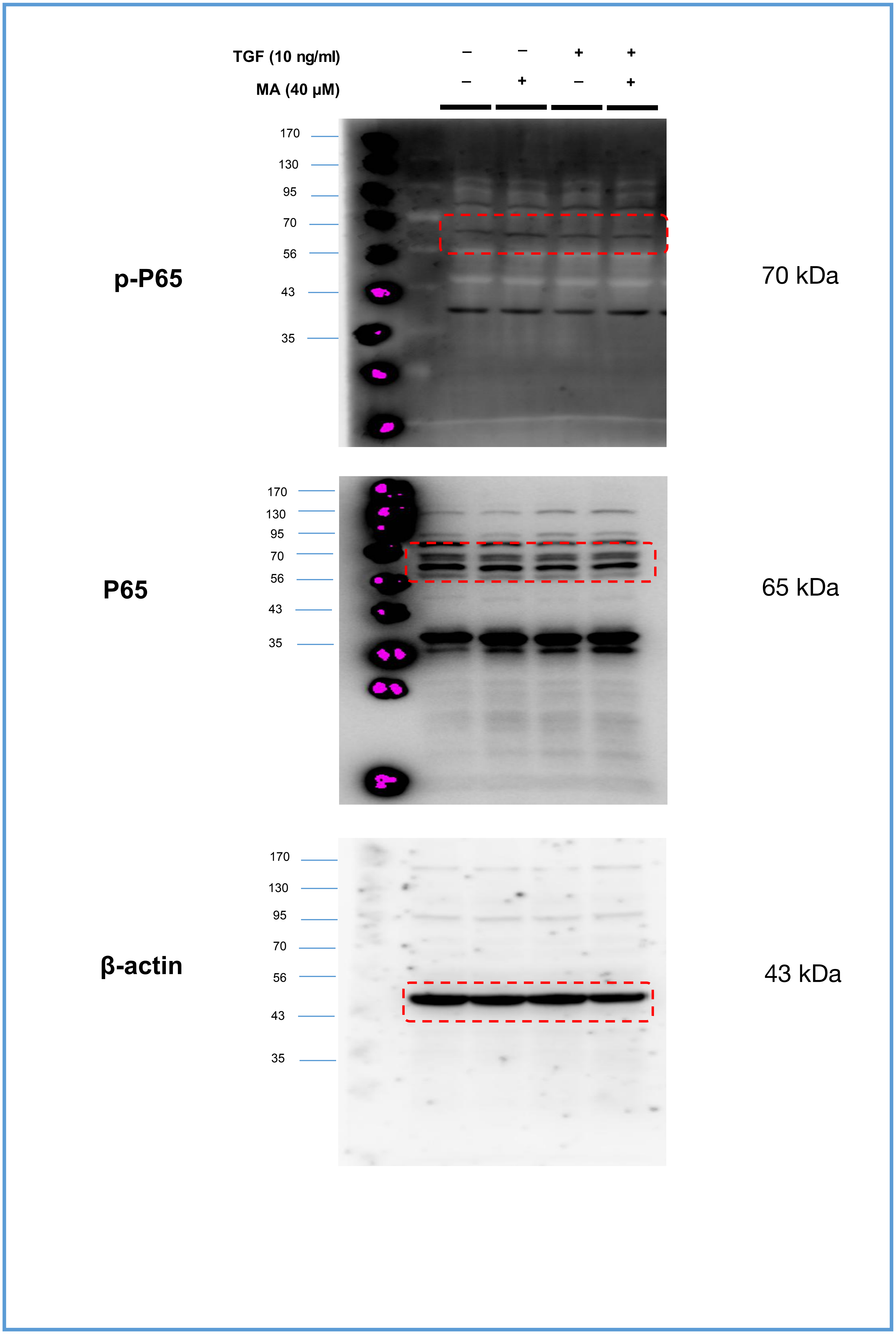

TGF (10 ng/ml) – – + +
MA (40 μM) – + – +
p-P65
170
130
95
70
56
43
35
70 kDa
P65
170
130
95
70
56
43
35
65 kDa
β-actin
170
130
95
70
56
43
35
43 kDa

Figure S2 C

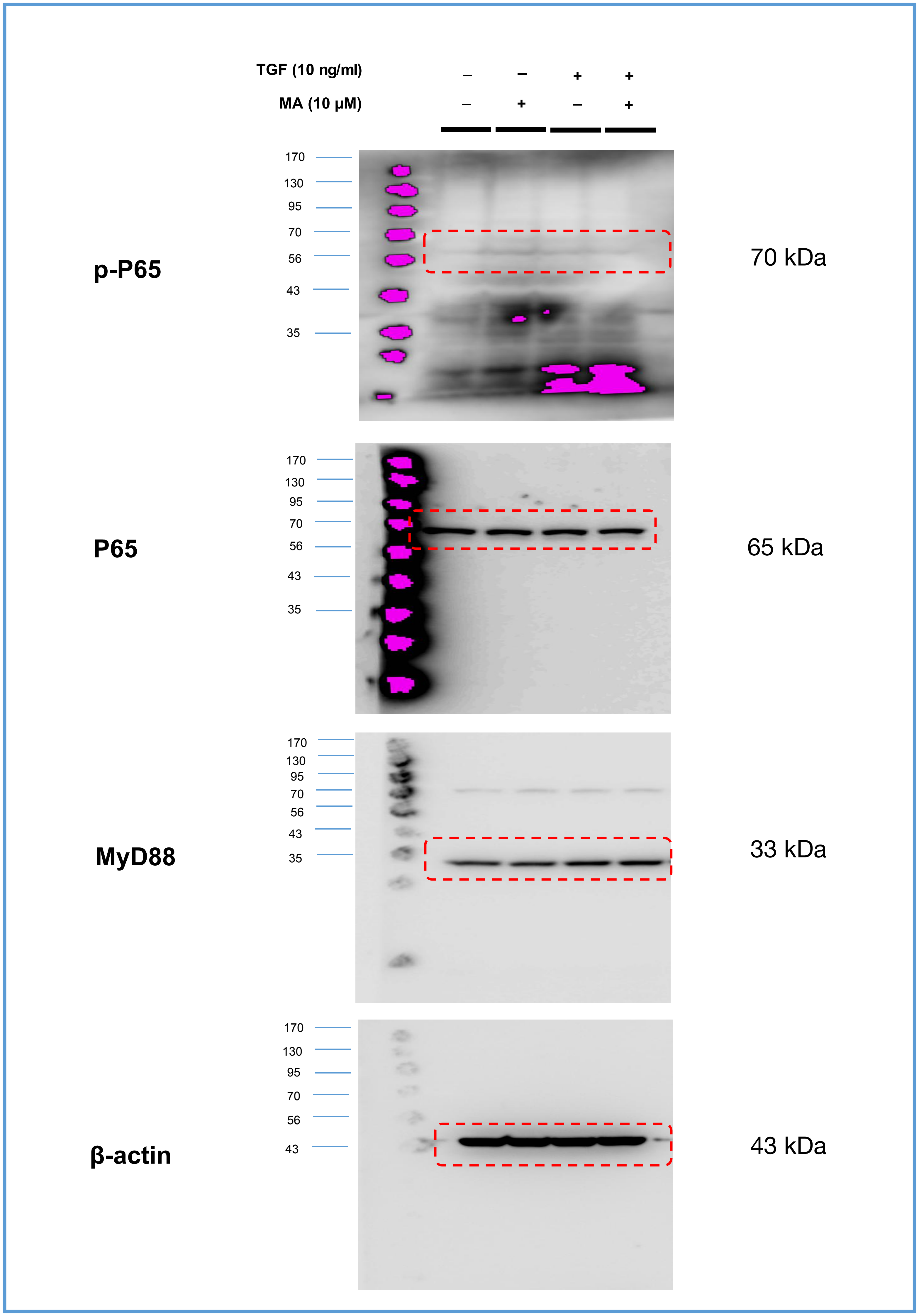

TGF (10 ng/ml) – – + +
MA (10 μM) – + – +
p-P65
70 kDa
P65
65 kDa
MyD88
33 kDa
β-actin
43 kDa
170
130
95
70
56
43
35
