# Supplementary material for: Renoprotective Effects of Maslinic Acid on Experimental Renal Fibrosis in Unilateral Ureteral Obstruction Model via Targeting MyD88
Source: Front Pharmacol. 2021 Sep 13;12:708575. doi: 10.3389/fphar.2021.708575 (PMC8475766; doi:10.3389/fphar.2021.708575)
Supplement: Supplementary file 2 [file DataSheet1.PDF]

Supplementary Materials

Renoprotective effects of maslinic acid on experimental renal fibrosis in unilateral ureteral obstruction model via targeting MyD88

Wenjuan Sun, Chang Hyun Byon, Dong Hyun Kim, Hoon In Choi, Jung Sun Park, Soo Yeon Joo, In Jin Kim, Inae Jung, Eun Hui Bae, Seong Kwon Ma, Soo Wan Kim*

Department of Internal Medicine, Chonnam National University Medical School, Gwangju 61469, Korea

* Correspondence to Soo Wan Kim, MD, PhD, Department of Internal Medicine, Chonnam National University Medical School.

42 Jebongro, Gwangju 61469, Korea

E-mail: skimw@chonnam.ac.kr

Tel.: +82-62-2206271, Fax: +82-62-2258578

Table of Contensts

Figure S1. MA decrease the mRNA expression of MyD88 in vivo and in vitro.

Figure S2. MA has no effect on the Nf-κB signaling in TGF-β stimulated NRK49F cells and NRK52E cells.

Table 1. Primers for RT-PCR

Figure legends

Figure S1. MA decrease the mRNA expression of MyD88 in vivo and in vitro.

(A) mRNA expression levels of MyD88 in kidney model was detected by by RT-PCR. n = 6. *P < 0.05, **P < 0.01, ns: no significance. (B) mRNA expression levels of MyD88 in TGF-β treated NRK49F cells were determined by RT-PCR. Cells were pretreated with MA for 2 h after starvation with 0.5% FBS medium, then treated with TGF-β (10 ng/ml) for 30 min. The data are presented as the mean ± SD, n = 3. *P < 0.05, **P < 0.01, ns: no significance. MA, maslinic acid; UUO, unilateral ureteral obstruction; RT-PCR, Real-Time PCR.

Figure S2. MA has no effect on the Nf-κB signaling in TGF-β stimulated NRK49F cells and NRK52E cells.

(A) Expression of p-P65 and P65 in TGF-β-treated NRK49F cells was detected by western blot and quantified by densitometry. Cells were pretreated with MA for 2 h after starvation and then treated with TGF-β (10 ng/mL) for 30 min. (C) Expression of p-P65 and P65 an MyD88 in TGF-β-treated NRK52E cells was detected by western blot and quantified by densitometry. Cells were pretreated

with MA for 2 h after starvation and then treated with TGF-β (10 ng/mL) for 30 min. (B,D,E) Statistical significance was presented as the mean ± SD, n = 3. *P < 0.05, **P < 0.01, ns: no significance.

Figure.S1

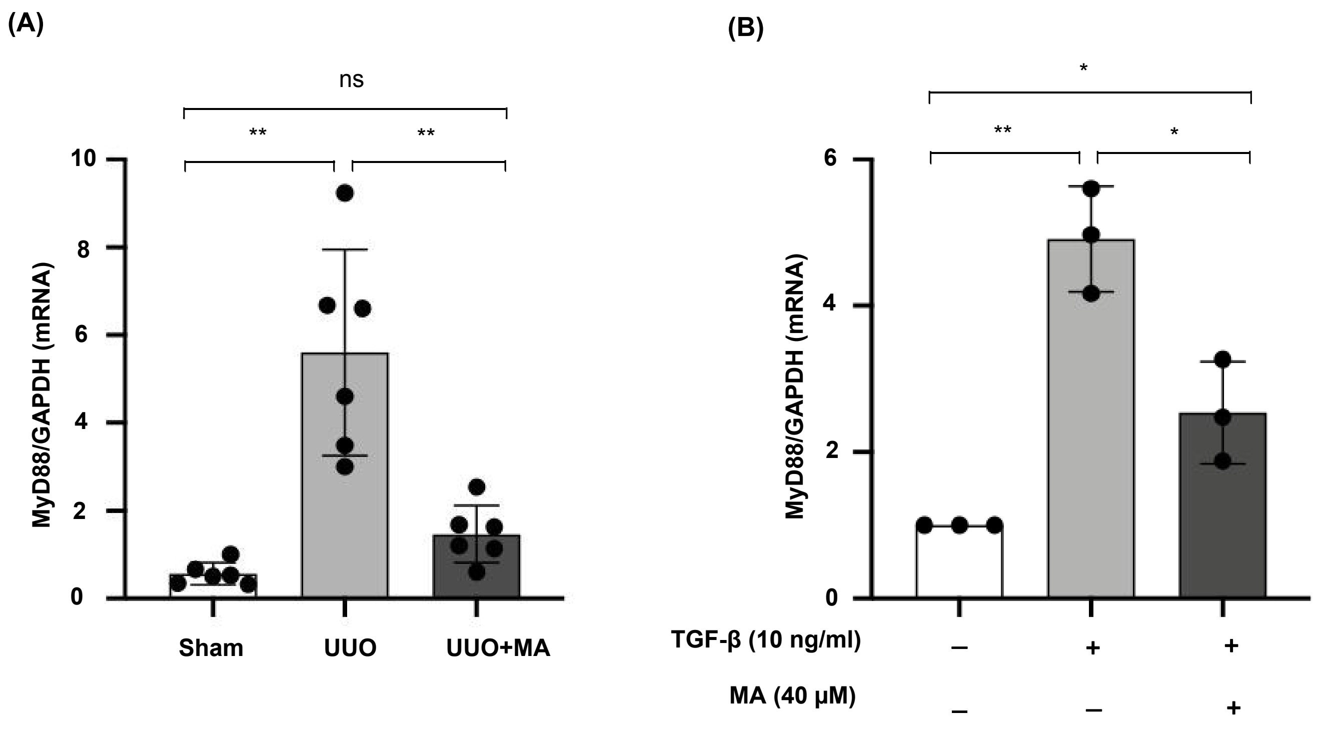

(A)
ns
**
**
10
8
6
4
2
0
MyD88/GAPDH (mRNA)
Sham
UUO
UUO+MA
(B)
*
**
*
6
4
2
0
MyD88/GAPDH (mRNA)
TGF-β (10 ng/ml)
–
+
+
MA (40 μM)
–
–
+

Figure.S2

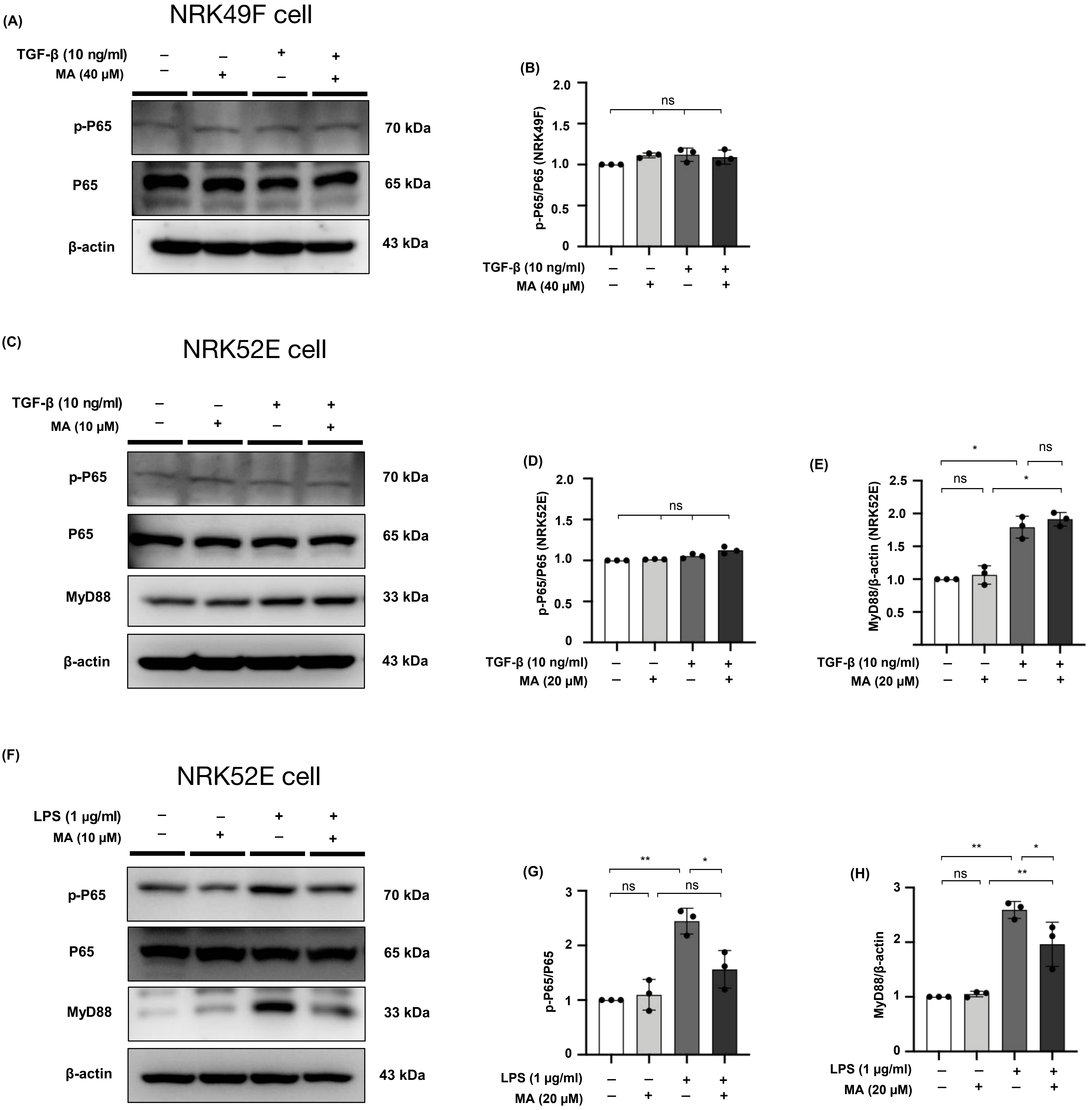
(A)
NRK49F cell
TGF-β (10 ng/ml)
MA (40 μM)
p-P65
70 kDa
P65
65 kDa
β-actin
43 kDa
(B)
p-P65/P65 (NRK49F)
ns
TGF-β (10 ng/ml)
MA (40 μM)
(C)
NRK52E cell
TGF-β (10 ng/ml)
MA (10 μM)
p-P65
70 kDa
P65
65 kDa
MyD88
33 kDa
β-actin
43 kDa
(D)
p-P65/P65 (NRK52E)
ns
TGF-β (10 ng/ml)
MA (20 μM)
(E)
MyD88/β-actin (NRK52E)
TGF-β (10 ng/ml)
MA (20 μM)
(F)
NRK52E cell
LPS (1 μg/ml)
MA (10 μM)
p-P65
70 kDa
P65
65 kDa
MyD88
33 kDa
β-actin
43 kDa
(G)
p-P65/P65
LPS (1 μg/ml)
MA (20 μM)
(H)
MyD88/β-actin
LPS (1 μg/ml)
MA (20 μM)

Table 1. Primers for RT-PCR

Gene	Primer sequence
Rat GAPDH	**F: 5′-GTCGTGGTCGTAGTGGGGTAAACTA-3′**<br>**R: 5′-GACTGTACGGCGGACCTCTTTGGAC-3′**
Mouse GAPDH	**F: 5′-TGTGTCCGTCGTGGATCTGA-3′**<br>**R: 5′-GATGCCTGCTTCACCACCTT-3′**
Rat MyD88	**F: 5′-CATACGCAACCAGCAGAAACA-3′**<br>**R: 5′-TCGTCAGAAACAACCACCACC-3′**
Mouse MyD88	**F: 5′-CCTACCCCCAGAAAAGAAGG-3′**<br>**R: 5′-CTGGGGAGAAAACAGCTGAG-3′**
Rat IL-1β	**F: 5′-TGATGTTCCCATTAGACAGC-3′**<br>**R: 5′-GAGGTGCTGATGTACCAGTT-3′**
Mouse IL-1β	**F: 5′-CAACCAACAAGTGATATTCTCCATG-3′**<br>**R: 5′-GATCCACACTCTCCAGCTGCA-3′**
Rat TNFα	**F: 5′-GTCGTAGCAAACCACCAAGC-3′**<br>**R: 5′- CTCCTGGTATGAAATGGCAA-3′**
Mouse TNFα	**F: 5′-GCATGATCCGCGACGTGGAA-3′**<br>**R: 5′- AGATCCATGCCGTTGGCCAG-3′**
Rat MCP-1	**F: 5′-ATCCCAATGAGTAGGCTGGAGAGC -3′**<br>**R: 5′-CAGAAGTGCTTGAGGTGGTTGTG -3′**
Mouse MCP-1	**F: 5′-CACCTGCTGCTACTCATTCACT-3′**<br>**R: 5′-GTTCTCTGTCATACTGGTCACTTCT-3′**
Rat ICAM-1	**F: 5′-GCCCGGAGGATCACAAACGAC-3′**<br>**R: 5′-CCTGGGGCTGGCATGTAAGAGT-3′**
Mouse ICAM-1	**F: 5′-CACCCCAAGGACCCCAAGGAGAT-3′**<br>**R: 5′-CGACGCCGCTCAGAAGAACCAC-3′**
